# Bioceramics for Hip Joints: The Physical Chemistry Viewpoint

**DOI:** 10.3390/ma7064367

**Published:** 2014-06-11

**Authors:** Giuseppe Pezzotti

**Affiliations:** 1 Ceramic Physics Laboratory, Kyoto Institute of Technology, Sakyo-ku, Matsugasaki, Kyoto 606-8126, Japan; E-Mail: pezzotti@kit.ac.jp; Tel./Fax: +81-757-247-568; 2 Department of Orthopedic Research, Loma Linda University, 11406 Loma Linda Drive, Suite 606 Loma Linda, CA 92354, USA; 3 The Center for Advanced Medical Engineering and Informatics, Osaka University, Yamadaoka, Suita, Osaka 565-0871, Japan; 4 Department of Molecular Cell Physiology, Graduate School of Medical Science, Kyoto Prefectural University of Medicine, Kamigyo-ku, 465 Kajii-cho, Kawaramachi dori, Kyoto 602-0841, Japan

**Keywords:** hip joint, ceramics, oxygen vacancy, alumina-zirconia composites, silicon nitride, cathodoluminescence spectroscopy

## Abstract

Which intrinsic biomaterial parameter governs and, if quantitatively monitored, could reveal to us the actual lifetime potential of advanced hip joint bearing materials? An answer to this crucial question is searched for in this paper, which identifies ceramic bearings as the most innovative biomaterials in hip arthroplasty. It is shown that, if *in vivo* exposures comparable to human lifetimes are actually searched for, then fundamental issues should lie in the physical chemistry aspects of biomaterial surfaces. Besides searching for improvements in the phenomenological response of biomaterials to engineering protocols, hip joint components should also be designed to satisfy precise stability requirements in the stoichiometric behavior of their surfaces when exposed to extreme chemical and micromechanical conditions. New spectroscopic protocols have enabled us to visualize surface stoichiometry at the molecular scale, which is shown to be the key for assessing bioceramics with elongated lifetimes with respect to the primitive alumina biomaterials used in the past.

## 1. Introduction

Evolutional processes of the human body, taking place through continual and unabated adaptations over a period of many millions of years, has led to optimization in both form and functions of diarthrodial joints. Among such complex structures, consisting of hard tissue, soft tissue, and fluids, the hip joint is the largest one in our body, and also a heavily demanded one for repetitive loads of high magnitude from daily activities. Under such severe conditions, any of the constituent parts of the hip joint might break down in its structure through sudden injury or degenerative diseases. Any breakdown might result in impaired joint mobility and pain. As the hip joint is particularly prone to failure, any such failure is typically treated through a surgical intervention, commonly referred to as total hip arthroplasty (THA). This is a quite invasive surgery and involves using artificial materials to replace the bearing surfaces of both femur (*i.e*., the femoral head component) and pelvis (*i.e*., the acetabular liner component). It should be emphasized at the outset that artificial biomaterials, despite being inherently stronger than biological tissues, could by no means surpass them in their biological response and lubrication capacity. Having said this, we should continuously search for bearing biomaterials with improved performance and elongated lifetime *in vivo*. One aspect of the general statements in focus here is to appraise how difficult could be to reproduce in full the characteristics of bioinertness and the efficient lubrication mechanisms of human joints. Artificial prostheses, indeed, unavoidably show a limited lifetime and remain insufficiently adjusted for operation in the human body, as compared to human joints.

Notwithstanding the foregoing, the great majority of THA surgeries have indeed proven quite successful, as far as most patients are satisfied with both the achieved pain reduction and increased mobility [1,2,3]. Moreover, some long established and widely used hip prostheses have experienced survival rates >90% after 10 years [4]. However, statistics teach us that a significant (and increasing) number of patients have to endure a revision surgery [5]. While such cases might highlight the fact that something is systematically missing in the overall THA protocol, we need to distinguish at the outset between: (i) revision surgeries occurring within two or three years from the primary surgery (*i.e*., before elapsing the expected lifetime of the hip prosthesis), thus due to poorly manufactured implants, poor design, or surgical errors; and, (ii) revision surgeries occurring at >10 years implantation and associated with the unavoidably limited lifetime of the implanted synthetic material.

Specifically regarding the above point (i), a considerable body of legalization has now been enacted to govern the medical device industry (e.g., including the so-called Medical Device Amendment Acts in the US and the Medical Device Directives in the European Union). The primary purpose of these laws and regulations is to ensure both safety and effectiveness of the marketed devices. These objectives are achieved through preliminary testing the medical devices (*i.e*., including a pre-clinical stage with laboratory bench tests and several clinical stages including randomized and multicenter clinical trials). Our past studies have only marginally been involved with type (i) failures, specifically with reference to particularly evident and massive cases of material failures such as the catastrophic fractures of acetabular cups in sandwich-type ceramic-on-ceramic hip joints [6] and of environmentally degraded ceramic femoral heads [7]. Nevertheless, we have clearly stated our ideas on the insufficiency of the protocols of quality control, in our opinion associated with a lack of pre-operative control (e.g., to be performed in the hospitals) for each individual joint implanted [8,9]. The introduction of such protocols, which we have proposed as being easily achievable through Raman spectroscopy, is particularly stringent for controlling the state of oxidation of polyethylene liners. Note that type (i) failures, somewhat erratic in their nature, cannot be suppressed or even reduced through merely enlarging on (or further complicating) the body of regulatory requirements. These types of failure have no relationship with biomaterial components matching or not the conditions and the properties declared in the preliminary regulatory process. On the contrary, there is a threshold for regulatory requirements beyond which the need for guarantying patients’ safety unavoidably encroaches upon *de facto* delaying the proposal of new products in the biomedical market. From a purely technological viewpoint, however, we find hardly conceivable how nowadays deterministic levels of quality control could even be applied in sub-micron-sized electronic devices [10], while they are yet conspicuously missing in macroscopic joint devices (*i.e*., despite the high impact of the latter ones on social welfare).

Regarding the above point (ii), the main issue resides in the amount of revision surgeries associated with lifetime expiration of what could be judged as a “successful” hip implant. In this latter context, a challenge to be taken up resides in the development of not only more reliable, but also more durable biomaterials. We have extensively discussed elsewhere our strategic view for future developments in the specific field of bioceramics [8,9]. In this paper, we shall further inquire into intrinsic biomaterial issues related to their “natural” cycle of lifetime when embedded in biological environment. In particular, we shall look into whether new *in vitro* experimental testing protocols could actually be devised, which enable differentiating the intrinsic lifetime performance of different hip prostheses. Such being the case, which testing protocols could prove the most appropriate? Our recent spectroscopic research suggests that the answer might lie in the physical chemistry of the biomaterial surfaces [11,12,13,14,15,16,17,18,19,20], the issue of much contention in pre-clinical experimental testing of hip prostheses thus resulting shifted toward the possibility of specifying additional (and more effective) evaluation criteria and protocols. In the opinion of this author, new protocols should additionally rely on designing and monitoring the bearing surfaces at the molecular scale. Design criteria shall newly be suggested here based on physical chemistry arguments.

## 2. Background on Physical Chemistry of Bioceramics

### 2.1. A serious Inquiry on Bioinertness of Ceramic Oxides

Long-standing definitions of bioinert materials include at top-list positions some ceramic oxides, such as alumina (Al_2_O_3_) and zirconia (ZrO_2_; or, if partially stabilized with 3 mol% yttrium oxide (Y_2_O_3_), commonly referred to as 3Y-TZP) [21,22]. Such oxide biomaterials have extensively been described as extremely stable in biological environment and endowed with long-term structural reliability (*i.e*., intended as the preservation of their pristine mechanical properties, as measured before *in vivo* exposure), in addition to their peculiar capability of eliciting a minimal response in the host tissues. These are indeed true and precious properties. Such excellent functional behavior definitely represents a fundamental requirement for a biomaterial to be employed in hip arthroplasty. However, any invoked definition of “long-term” performance of biomaterials for hip joints will clearly be a matter of bioinertness definition, of the scale at which bioinertness is monitored, and of lifetime expectation as well.

The selection of biomaterials for artificial joints is heavily constrained by conventional concepts of mechanical engineering, mainly underlying their fracture strength, reliability, and tribological response. During the last decade, however, the requirements from patient side appear to have undergone a quite radical evolution towards additional and more demanding tasks. In other words, as extended lifetime expectations for arthroplastic patients represent nowadays the new main target, a fundamental process of revalidation becomes needed for establishing the new criteria upon which joint design and biomaterial choices should be based. Specifically, in relation to surface engineering concepts in joint arthroplasty, a paradigmatic shift is required to establish new evaluation criteria taking into account the pivotal role of surface reactivity and related off-stoichiometry (evolutional) characteristics of the joint bearing surfaces during service operation. While the most recent studies of advanced polyethylene liners have brought revitalized attention to the pivotal role of oxidation chemistry on the expected lifetime of the implanted joints, the “chemistry” approach is yet conspicuously missing for ceramic components, as they are erroneously considered to be completely bioinert. In reality, the situation is not quite so straightforward. If the bearing surfaces of hip joints should preserve their mechanical and physical characteristics for quite a long-term span of time despite the interference from an extreme (*i.e*., cumulatively in terms of mechanical, thermal, and chemical loading) physiological environment, a finer tuning of their molecular structure should be mandatorily required. We shall show in the remainder of this paper how fundamental features related to the inherently ionic structure of ceramic oxides and to the complex nature of the biochemical environment of hip joints could severely exacerbate surface degradation processes. In other words, as far as hip implants are concerned, the definition of bioinertness should mandatorily encompass not only a macroscopic view of retaining the bulk integrity of the biomedical implant, but also the resistance to degradation of its surface at the molecular level. This latter is indeed a property extremely difficult to achieve. Even very stable materials as oxide ceramics interact at their surface to some extent with the biological environment, such interaction locally altering their inherent properties in terms of hardness, wear resistance and toughness. We shall thus start our discussion from first accepting the concept of “impossibility of inertness”, as already stated by Williams [23], and extending it to the molecular scale. Accepting such impossibility is perhaps the most appropriate starting point for studying the bearing surfaces of artificial hip joints.

### 2.2. Oxygen Sub-Lattice in Bioceramic Oxide Surfaces

The nascent surfaces developed upon wear in artificial hip joints are extraordinarily chemically active, with a variety of tribochemical reactions becoming energetically active there [24,25]. Hydrothermal environment on the surface of Al_2_O_3_ bearings is expected to lead rather to dissociation than to adsorption [26]. This process is exacerbated by the fact that Al_2_O_3_ biomaterials are polycrystalline in nature, and thus they incorporate an internal network of grain boundaries, namely off-stoichiometric and crystallographically faulted locations in the biomaterial structure. The phenomenon of dissociation produces hydroxyl and proton radicals, which in turn promote the formation of surface vacancies for preserving electrical charge neutrality [14,27]. A major obstacle to a rigorous understanding of the “intrinsic” surface properties of alumina ceramics resides in correctly locating at the atomic level the ubiquitous presence of hydrogen [28,29,30,31,32,33,34,35,36,37,38,39]. Molecular H_2_O and surface hydroxyl groups are generally present because of the adsorption of H_2_O from the environment [39,40,41,42,43,44,45,46,47,48,49,50,51,52,53]. It has rigorously been established that the low coordination of surface Al atoms, present on the surface of grains closely oriented as (0001) corundum planes, makes those sites becoming strong electron acceptors. Such atomic sites thus readily adsorb H_2_O molecules through oxygen atoms. At low coverage regimes, H_2_O prefers to dissociate, but can also adsorb in a metastable molecular binding mode. Dissociative adsorption is more favorable than molecular adsorption and is primarily heterolytic in nature. This means that adsorbed H_2_O can be viewed as splitting into H^+^ and OH^−^, with the proton transferred to a nearby surface O [54]. The solid state chemical reaction at equilibrium on alumina surfaces in hydrothermal environment can thus be written as:

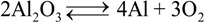
(1)

This is in turn associated with the elementary reaction:

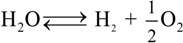
(2)

Elam *et al.* [40] acknowledged the presence of oxygen vacancies in discussing the values of kinetic parameters obtained from modeling the hydroxyl coverage of (0001) sapphire surfaces. In frictional sliding of alumina surfaces, even under ideal sliding conditions (*i.e*., without including third-body wear or microseparation processes) [55,56], continuous formation, dissociative adsorption, and frictional removal of hydroxylated layers should be expected to repeatedly create and annihilate a large population of different kinds of vacancy site in the outer surface layers of alumina lattice, according to mechanochemically activated processes. Moreover, the well-known hydrophilicity of Al_2_O_3_ sliding surfaces should, in principle, minimize any surface adsorption of proteins, and thus their consequent denaturation and irreversible unfolding [57,58,59]. However, a scarcity of synovial fluid lubricant in ceramic self-mating hip joints might create an extremely severe thermomechanical environment [60], in which modifications of the alumina lattice (*i.e*., especially in correspondence of grain boundaries) might increasingly take place. In this context, one could also hypothesize a role of protein by-products (e.g., hydrocarbons [61]) or body-released ions (e.g., Ca^2+^, Mg^2+^, and Na^+^) on possible stoichiometric alterations of the alumina lattice. It is known, for example, that dilution of sodium hyaluronate (with release of Na^+^ ions) takes place in patients affected by rheumatoid arthritis, as systematically detected by Dahl *et al.* [62]. Although the effect of the above tribochemical factors on the wear behavior of alumina bearings has not yet been discussed in details, we shall show in the remainder of this paper some evidence not only for vacancy formation (*i.e*., for a lack of bioinertness at the molecular scale), but also for a clear interaction between ions released from the body environment and the alumina lattice. The ensemble of various tribochemically driven events should give, in the long term, a contribution far from marginal to the lattice structure of alumina bearing surfaces, thus also altering their mechanical and tribological resistance. The challenge is thus that of linking the new body of independent experimental evidence, which reveals a complex cascade of chemical events (*i.e*., from the formation of point defects to the occurrence of plastic flow in the lattice), to the *in vivo* wear performance of monolithic alumina.

The dispersion of stoichiometric (point) defects in Y-TZP systems depends on both dopant concentration and surface segregation phenomena [63]. In the bulk, the yttrium dopant tends to form a pair (*i.e*., two yttrium atoms close to each other) and preferentially occupying first or second nearest neighboring sites to the compensating oxygen vacancy [64]. On the other hand, at the surface, yttrium tends to be segregated in the top layers (up to 4~5 Å) of the dominant (111) surface of Y-TZP. The composition of the outermost surface of Y-TZP is predicted to be independent of Y bulk concentration and reach a maximum Y/Zr ratio of 1:1. This circumstance involves the maximum concentration of oxygen vacancies being located at the top surface of the bearing component. In general, the formation of oxygen vacancies by yttrium doping provides active sites for oxygen adsorption. In addition, the low-coordinated Zr cations on the Y-TZP surfaces can attract strongly reduced oxygen species, and the most stable adsorption state of oxygen might enable the surface to achieve a higher bond saturation of the neighboring Zr and the volume expansion associated site. The tetragonal phase of ZrO_2_ in its stoichiometric state is only stable at high temperature with the tetragonal-to-monoclinic transformation unavoidably introduces large residual stresses in the microstructure [13,17]. It is known that room-temperature stabilization of tetragonal zirconia can be achieved by addition of large substitutional cations (*i.e*., isovalent to Zr^4+^; e.g., Ce^4+^) to expand the lattice volume, or by doping with subvalent cations to create oxygen anion vacancies (e.g., Y^3+^), or by a combination of these two effects [65]. The formation of oxygen vacancies serves to locally reduce the average coordination number and, through alleviating oxygen overcrowding around the Zr^4+^ ions, to facilitate the relaxation of the oxygen sub-lattice towards a cubic symmetry. The vacancy mechanism, which is known to be more effective than simply expanding the lattice volume, is expressed by the following equilibrium equation:

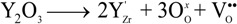
(3)
where, 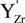 represents the Y^3+^ ions on regular cationic sites (*i.e*., replacing Zr^4+^ ions) associated with a relative charge of −1; 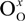 stands for oxygen ions on regular anionic sites and 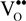 represents the double positively charged (with respect to the charge of regular oxygen ions) oxygen vacancies. The above notation is referred to as the Kröger-Vink notation for lattice elements and point defects in crystal structures [66]. This formalism is useful to concisely describe the atomistic reactions related to the replacement of Zr^4+^ by Y^3+^. Note that two ions of Y-dopant are necessary to electrically balance a single oxygen vacancy. Accordingly, the concentration of vacancies in the anionic sublattice can, in principle, be calculated from the concentration of Y-ions at cationic sites and might easily reach the range of few percent, as follows:

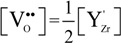
(4)

The concentration of intrinsic defects thermally induced during sintering is, in contrast, negligible in the usual range of manufacturing temperatures, which explains the full instability of pure zirconia in its tetragonal phase at room temperature. The peculiar configuration of oxygen vacancies and sub-valent Y-ions has the effect of reducing the average coordination number of the zirconium atoms from 8 (*i.e*., as in the cubic structure) to values closer to 7 (*i.e*., similar to the monoclinic phase), thus stabilizing the tetragonal phase at room temperature. The surface structure of ZrO_2_ lattices imparts crucially important characteristics to the performance of a load-bearing biomaterial. Accordingly, experimental techniques and computational modeling have extensively been applied to investigate surface structures and properties of zirconia ceramics. One aspect particularly important here should thus be the difference between surface and bulk stoichiometry, as already mentioned above. In polycrystalline materials, surface segregation and, thus, charge of the compensating oxygen (anion) vacancies are likely to experience substantial gradients in space not only within the bearing surface but also below it, with surface chemistry and stoichiometry effects dominating the statistical presence of active sites for the formation of metastable nuclei *in vivo*.

According to the above considerations, it is clear that the stoichiometry of zirconia (especially at its free surface) plays a fundamental role in phase stability and, thus, in the overall performance of the material in bearing components. While this peculiarity has indeed offered a wide range of material design opportunities in many different fields of application, it also forewarns technologists wanting to use zirconia as a structural biomaterial. The human body certainly represents an extremely active chemical environment, in which oxygen vacancies can be easily annihilated by protons and/or cations replaced, especially due to the combined effects of chemical and strain gradients between surface and subsurface. It should be thus very surprising if chemically “susceptible” ceramic compounds, such as the family of partly stabilized zirconia materials, could preserve unchanged upon long-term *in vivo* exposures the same stoichiometry established at the time of material fabrication. If even an extremely stable oxide like alumina undergoes substantial stoichiometric changes in the severe tribochemical environment of human hip joints, one could easily imagine that also the less stable zirconia lattice, when embedded in the human body, will necessarily change its chemical state and, consequently, its crystallographic structure. These changes are accelerated by the presence of mechanical stresses and thus preferentially start from the very surface of the bearing components. Again, full bioinertness can hardly be found. Polymorphic transformation in tetragonal ZrO_2_ will necessarily occur in a hydrothermal environment, it is just a matter of time.

### 2.3. Monitoring Surface Off-Stoichiometry on Hip Bearing Surfaces

**Figure 1:**
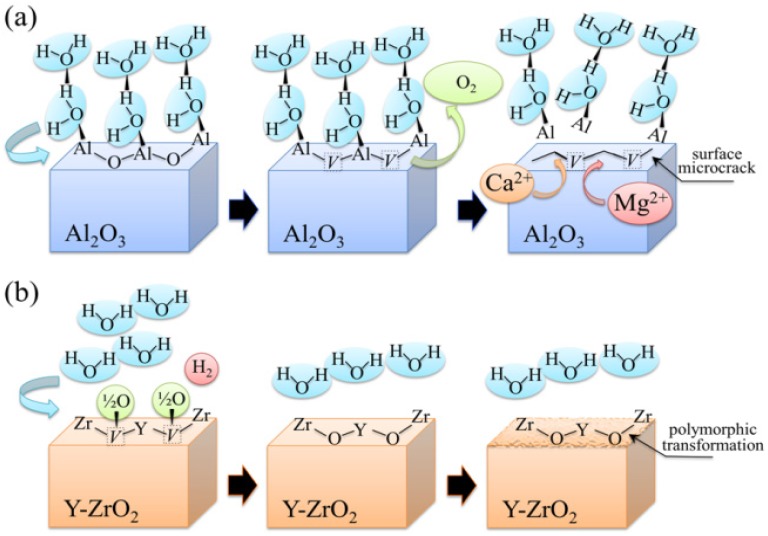
Drafts of the different behaviors of (**a**) alumina and (**b**) zirconia bioceramic surfaces in a biologically active environment with emphasis placed on their oxygen-sublattice activity.

It has been discussed in the previous section how the behavior of alumina and zirconia ceramics in a biologically active environment is quite different, if not even opposite, in terms of oxygen-sublattice activity. These different behaviors are schematically drawn in Figure 1a,b, respectively. Alumina tends to give up oxygen from the structure of its surface as a consequence of hydroxylation processes, thus creating an increasing vacancy concentration in its lattice with progressing hydrothermal exposure. Such state of substantial off-stoichiometry then triggers the possible incorporation of sub-valent cations (*i.e*., Ca^2+^, Mg^2+^, and Na^+^) from the joint environment under tribochemical loading. On the other hand, partially stabilized zirconia, 3Y-TZP, already containing a wealth of oxygen vacancies as a consequence of intentional doping by aliovalent yttria (*i.e*., for the purpose of its (partial) stabilization), tends to fill those vacancies with adsorbing and incorporating in its structure the oxygen coming from the biological environment. Bearing in mind such peculiar difference, the purpose of this section is twofold: (i) to briefly describe the expected impact of those different stoichiometric behaviors on surface residual stresses at the microscopic scale (the effect of such stresses will be substantiated later in this paper by showing wear resistance data); and, (ii) to introduce a method for directly (and promptly) visualizing off-stoichiometry characteristics in the ceramic bearing surfaces, which opens the way to experimental demonstrations of the above point (i).

In the context of the above point (i), the first question coming to mind for alumina bearings could be: where does the oxygen that the surface releases during service operation actually go? The fact that there is no direct toxicity involved with such release does not mean that there are also no negative consequences in the long-term functionality of the joint. However, one needs here to make a distinction between hard-on-soft and hard-on-hard bearings. In the former case, the strong intrinsic affinity shown by polyethylene to be oxidized owing to the unavoidable presence of free radicals in its structure might suggest that bearing surfaces prone to release oxygen (e.g., Al_2_O_3_) should not, in principle, be “polyethylene friendly”. On the other hand, the formation of oxygen vacancies in hard-on-hard bearing surfaces represents just the beginning of a physicochemical chain reaction leading to local degradation of the mechanical properties of the joint surface, as it shall experimentally be demonstrated later in this paper. One should thus expect a reduced wear resistance, with possible grain detachment, with progressing oxygen vacancy formation and the successive incorporation of sub-valent cations (*i.e*., Ca^2+^, Mg^2+^, and Na^+^) into the faulted surface structure of the alumina lattice.

**Figure 2:**
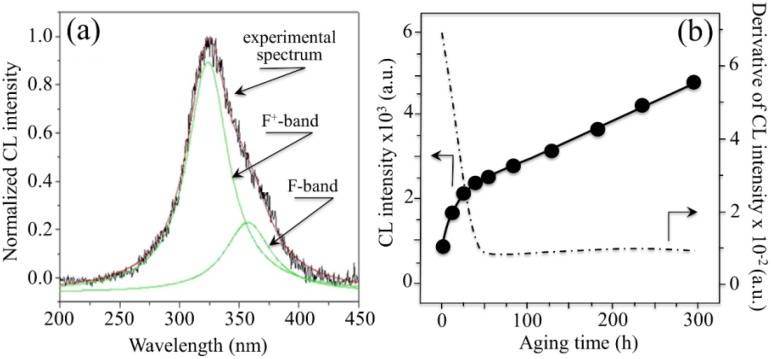
(**a**) A typical cathodoluminescence (CL) spectrum of biomedical alumina. Deconvolution is made into two Gaussian bands of F^+^ and F centers; (**b**) evolution of CL intensity (or oxygen-vacancy concentration) upon exposure time in hydrothermal environment of biomedical Al_2_O_3_ in an autoclave operating at 121 °C under 2 bar pressure of water vapor. The size of the used symbols corresponds to the standard deviation of the measured CL intensity. The derivative of the curve is also shown, which represents the *in vitro* vacancy-formation rate on the bearing surface.

Regarding the stability of 3Y-TZP bearing surfaces in hard-on-soft hip couples, oxygen incorporation to fill pre-existing vacancies could, in principle, be considered as a “polyethylene friendly” process. As a matter of fact, if oxygen “feels” more affinity to fill up the sub-stoichiometric lattice of zirconia, it will not participate or just participate with a reduced activity to the oxidation processes taking place on the polyethylene side of the joint. However, the annihilation of vacancy concentration at the surface of 3Y-TZP unavoidably leads to polymorphic transformation from the tetragonal to the monoclinic phase [64,65,66,67,68]. If transformation progresses toward the sub-surface of the zirconia femoral head, the local volume expansion and surface roughening involved with it might negatively affect the joint lifetime for a different reason: the formation of polyethylene debris due to enhanced abrasive wear. The impact of polymorphic transformation in hard-on-hard bearing couples made of 3Y-TZP has been found so destructive by *in vitro* simulation tests [69,70] that none of such combinations has even been proposed for commercial purposes. As far as the above-mentioned purpose (ii) is concerned, attention shall be drawn upon one powerful analytical tool in investigating surface off-stoichiometry of ceramic biomaterial surfaces, namely spatially and spectrally resolved cathodoluminescence (CL) spectroscopy. The basic principle of CL spectroscopy, which we apply here, can be simply described, as follows. If point defects are irradiated with electrons, they emit light at characteristic wavelengths. Such light emission cannot be observed if an unfaulted lattice is equally irradiated. Thus, the CL spectrum emitted by Al_2_O_3_ ceramics contains important information about the oxygen stoichiometry of the lattice [71,72,73]. In the ultra-violet region, a doublet consisting of partly overlapping bands can be found. One of the two bands is the so-called F^+^-center band, which is emitted by a single oxygen vacancy trapping one electron. It is located at around 325 nm (3.8 eV). The other band composing the doublet is a far less intense one, and it is commonly referred to as the F-center band (single oxygen vacancy trapping two electrons). This latter band is located at around 415 nm (3.0 eV). The presence of the F^+^/F oxygen-vacancy doublet is a common feature in the CL spectrum of any alumina. However, different intensities of it (*i.e*., for exactly the same irradiation conditions) represent different vacancy concentrations, the higher the intensity the higher the vacancy concentration. In Figure 2a, a typical CL spectrum of biomedical (*i.e*., high-purity) alumina femoral head (BIOLOX^®^*forte*, CeramTec GmbH, Plochingen, Germany) is shown. Deconvolution is made into two distinct Gaussian bands (*i.e*., the F^+^ and F bands). Oxygen vacancy centers are Schottky-type defects that act as electric charge compensators. Therefore, they could form (or annihilate) on the material surface as a direct consequence of interactions between the sample surface and the environment. In other words, the CL intensity observed in the ultra-violet region directly reflects oxygen-vacancy concentration, as vacancies stem from a sub-stoichiometric Al_2_O_3_ lattice. This spectroscopic circumstance opens the way to monitor the evolution of oxygen-vacancy concentration upon exposure to *in vitro* hydrothermal environment and then to apply such knowledge to the analysis of alumina prostheses after exposure *in vivo*. Figure 2b shows the evolution of CL intensity (*i.e*., oxygen-vacancy concentration) upon exposure time in hydrothermal environment of an as-received Al_2_O_3_ femoral head (BIOLOX^®^*forte*) in an autoclave operating at 121 °C (under 2 bar pressure water vapor environment). Exposures lasted for periods of time ranging between 0 and 300 h. CL investigations were always carried out immediately after removing the sample from the autoclave at the end of the duration of the test in hydrothermal environment. This latter figure is actually the proof that alumina ceramic is not fully bioinert and that the processes of hydroxylation and vacancy formation actually occur (as described in Figure 1a) on its surface. The surface is thus driven toward increasingly pronounced off-stoichiometric states. Taking the derivative of this plot (also shown in Figure 2b) somewhat reduces the qualitative nature of the plot with giving the rate of vacancy formation in hydrothermal environment. Another important peculiarity of the CL band from F^+^ sites is the dependence of its wavelength at maximum on mechanical stress. Such dependence has been rigorously proved and calibrated, and it has been shown that wavelength shifts are directly proportional to the trace of the stress tensor, σ_*ii*_, stored in the portion of material irradiated by the electron probe [74,75]. These findings opened the way to use oxygen vacancies as stress sensors to monitor the residual stress state stored onto the surface of hip bearing components.

We also anticipate that additional CL bands in Al_2_O_3_ might arise from the presence of aliovalent cations in the lattice (*i.e*., already present as impurities or entering the surface lattice of Al_2_O_3_ during *in vivo* exposure). Divalent cations (e.g., Ca^2+^ and Mg^2+^) might substitute for Al^3+^ forming a complex F_c_-center, namely an F^+^-center adjacent to a divalent cation site. Emission from F_c_-centers of divalent cations produces distinct CL bands, located in a relatively narrow wavelength interval at around 300 nm [73]. Moreover, a doublet emission in the wavelength interval 450~600 nm of the CL spectrum was found to correspond to the formation of an MgAl_2_O_4_ spinel phase with higher ductility than Al_2_O_3_ [76].

**Figure 3:**
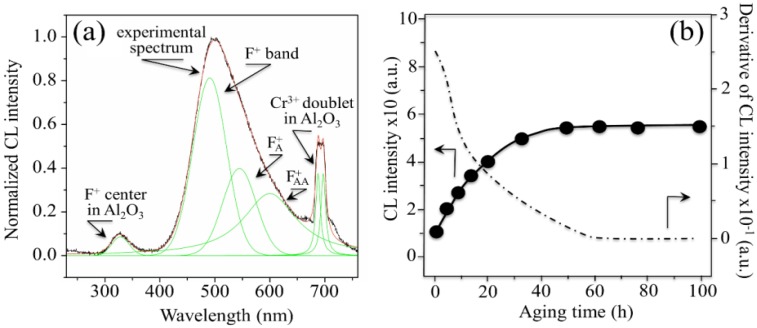
(**a**) Typical CL spectrum of biomedical 3Y-TZP. Three distinct emissions from oxygen-related defects with different structures overlap in the CL spectrum of ZrO_2_, namely the bands referred to F^+^, F_A_^+^, and F_AA_^+^ centers. Two extrinsic CL emissions also arise from the presence of Al_2_O_3_ dopant in biomedical 3Y-TZP; (**b**) evolution of CL intensity (or oxygen-vacancy annihilation) upon exposure time in hydrothermal environment of biomedical 3Y-TZP in an autoclave operating at 121 °C under 2 bar pressure of water vapor. The size of the used symbols corresponds to the standard deviation of the measured CL intensity. The derivative of the curve is also shown, which represents the vacancy annihilation rate on the surface.

The CL spectrum of 3Y-TZP (Figure 3a) features several bands overlapped to form a main broad emission. The most plausible interpretation foresees the sum of three distinct emissions from oxygen-related defects with different molecular structures [77]. Accordingly, the broad band that peaks at around 500 nm has been deconvoluted into three distinct sub-bands of Gaussian nature located at around 460 nm (2.69 eV), 550 nm (2.25 eV), and 600 nm (2.07 eV). The emission at 460 nm (commonly referred to as the F^+^-center of ZrO_2_) has been attributed to singly occupied anion vacancies, thus involving the presence of an intrinsic defect where all Zr^4+^ ions are nearest neighbors to the vacancy [78]. The two extrinsic bands centered at around 550 and 600 nm (*i.e*., referred to as F_A_^+^ and F_AA_^+^ centers, respectively) have been associated to electron transfer from the valence band to local mid-gap states. Their structures show one and two Y^3+^ ions as nearest neighbors to the vacancy, respectively [77,79]. While the intensity of the CL emission is unequivocally related to the presence of vacancy sites in the ZrO_2_ lattice, the efficiency of the CL emission is strongly affected by the local lattice configuration in the neighborhood of the point defect. The main consequence of this dependence also represents the most intriguing aspect of the CL emission of ZrO_2_: vacancy annihilation (rather than vacancy formation) leads to an increase (rather than to a decrease) of CL intensity. In a lack of direct experimental proofs, this experimental evidence might be interpreted in various ways. One way is by considering that the increase in CL intensity associated with oxygen vacancy annihilation is simply a consequence of the increased Zr-O bond stretching involved with oxygen incorporation. Such additional strain enables a more efficient emission as compared to relaxed oxygen-vacancy sites [79,80]. Another way to see the phenomenon is to foresee oxygen vacancies acting as charge traps and suppressing the charge and possibly also energy transfer. It should be noted that he electron beam creates high-energy excitations in the crystal structure, and relaxation of these excitations results in the appearance of electrons in the conductivity band and holes in the valence band. This relaxation is similar to that obtained under band-to-band excitation, but it is expected that under the highly energetic excitation of the electron beam the totality of the luminescence centers could be activated. Note also that excitation by laser within the band-gap directly excites the defects and thus results in an increase in luminescence emission for those same samples for which a decrease in CL emission is recorded [80].

Under electron beam excitation, electrons and holes are created, and the energy and/or charge transfer process takes place first. A decrease in CL intensity under electron excitation could thus simply mean that additional oxygen vacancies actually disturb the energy transfer to the defects responsible for luminescence. Possibly, oxygen vacancies act as charge traps, thereby decreasing the number of available electrons, the more numerous the vacancy sites the lower the CL intensity.

According to the peculiar behavior of zirconia with respect to its CL emission, a plot is given in Figure 3b of the dependence of CL intensity as recorded on the surface of an unused 3Y-TZP femoral head (NGK, Komaki, Japan; manufactured in 2007) upon increasing times of autoclave exposure (*i.e*., under the same conditions described above for Al_2_O_3_). In this plot, also the rate of vacancy annihilation in hydrothermal environment is shown. In addition to the CL emission related to oxygen vacancies, the presence of Si impurity in the ZrO_2_ lattice produced an additional band in the blue region of the CL spectrum (*i.e*., corresponding to the shoulder observed at around 390 nm (3.18 eV) [81]. Regarding the stress dependence of the CL spectrum of 3Y-TZP, unfortunately, no definite proof for such dependence could yet be obtained, mainly because of the strong overlapping of different sub-bands. Therefore, unlike the case of Al_2_O_3_, at this time it is not possible to obtain a quantification of residual stresses in zirconia polymorphs with directly using oxygen vacancy sites as stress sensors.

Finally, it should be emphasized that the CL probe has proved shallow to a nanometer scale [82,83] and, thus, capable to resolve both chemical and mechanical features at the very surface of ceramic biomaterials, as they develop upon both *in vivo* and *in vitro* environmental exposure. Drawing upon what has been learned about basic CL studies of oxide bioceramics, similar analyses were also performed on *in vivo* exposed alumina hip surfaces [8,14,83]. Monitoring could be concurrently obtained for the concentration of point defects (*i.e*., oxygen vacancy formation, their annihilation, the incorporation of substitutional impurities, and the formation of interstitial aluminum) trapped in the very neighborhood of the alumina surface and for lattice strain at the bearing surface, with a spatial resolution on the nanometer scale. CL tribochemical and micromechanical information then merged into a comprehensive picture of a crystallographic lattice affected by characteristics of extreme off-stoichiometry and by high strain fields.

In the remainder of this paper, we review some physical chemistry evidence obtained from both *in vitro* and *in vivo* analyzed ceramic hip joints, which encompasses relevant surface off-stoichiometry issues in Al_2_O_3_, ZrO_2_, and related composite biomaterials. As yet unexplored connections will be discussed as they become established between the wear resistance of such biomaterials and environmental factors peculiar to the human body.

## 3. Experimental Evidence and Future Strategies

### 3.1. The Limits of Monolithic Bioceramics

#### 3.1.1. Al_2_O_3_ Bioceramics

**Figure 4:**
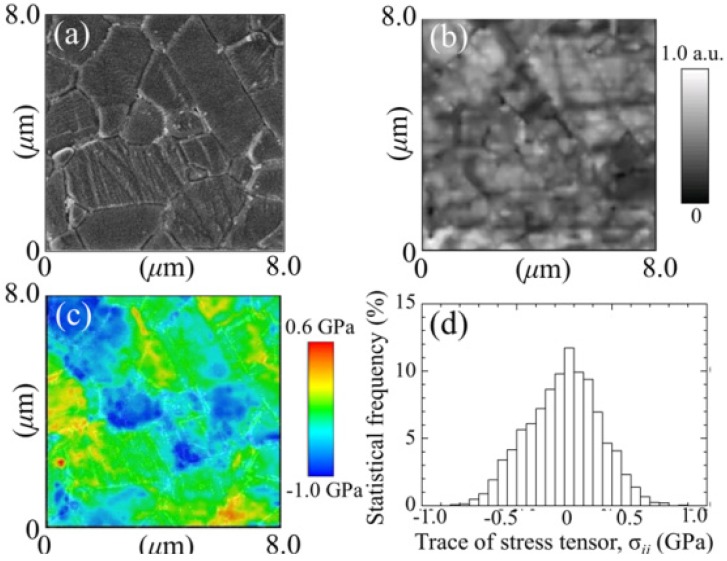
(**a**) A conventional scanning electron micrograph; (**b**) a CL intensity image; (F^+^/F band at 325 nm); and (**c**) a map of the trace of the residual stress tensor, σ_*ii*_; are shown for a randomly picked location on the surface of an early generation biomedical grade of polycrystalline alumina after long-term exposure *in vivo* (in a mildly worn area but away from the main wear zone); In (**d**), a histogram of the stress-trace magnitude is given, which represents the same data shown in the map (**c**). Stress magnitudes were deconvoluted in space, according to the procedure given in Refs. [75,82,83].

**Figure 5:**
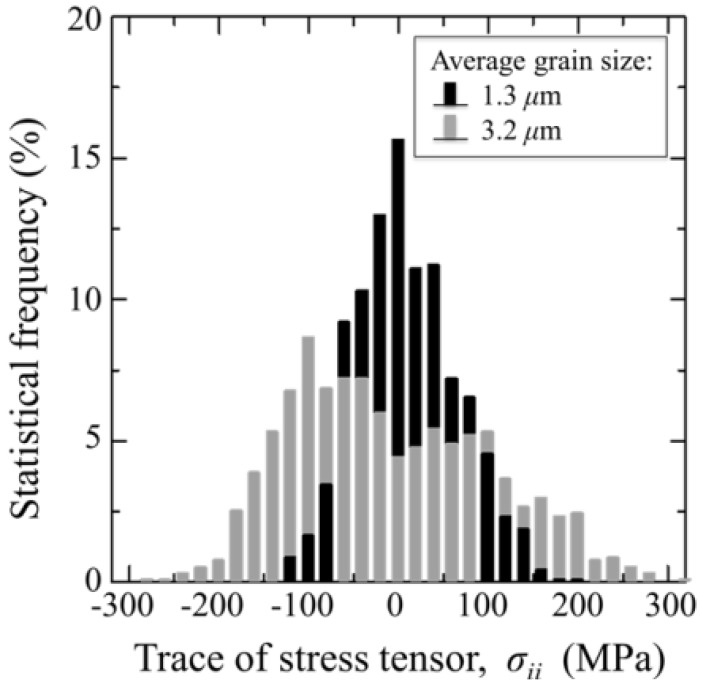
Average stress histograms collected on statistically meaningful surface areas of unused biomedical Al_2_O_3_ materials belonging to two different generations of Al_2_O_3_ bioceramics for hip joints. The average grain sizes are shown in inset.

We have mentioned above that grain boundaries in alumina polycrystals are both preferential locations for oxygen vacancies (and other point defects) and for intensification of residual stresses developed upon cooling from manufacturing temperature. Their faulted structure is a natural consequence of a (statistically) poor degree of coherency, as one could expect from grain-boundaries in a hexagonal structure (e.g., less symmetric than a tetragonal or a cubic one). On the other hand, a high magnitude of residual stresses arises from the high degree of anisotropy between thermal expansion coefficients along the *c*- and *a*-axis of the corundum structure. Both these circumstances have amply been documented in the literature both from theoretical and experimental sides [84,85,86,87,88,89,90]. Exacerbations of both phenomena could conceivably be expected due to hydrothermal exposure of the biomaterial surface. However, direct evidence at the nanometer scale is yet seldom. Figure 4 shows the usefulness of CL spectroscopy in visualizing stoichiometry and residual stress at the microstructural scale in polycrystalline Al_2_O_3_. In Figure 4a–c, a random location on the surface of an early biomedical grade of long-term *in vivo* exposed polycrystalline alumina (mildly worn but away from the main wear zone) was monitored by means of conventional scanning electron microscopy, by collecting a CL intensity image (F^+^/F band at 325 nm) and a map of the trace of the residual stress tensor, σ_*ii*_, on the material surface, respectively. No special preparation procedure was applied before CL analysis. Only a light coating with evaporated carbon was made on the sample to avoid charge up in the scanning electron microscope. This procedure will also apply to all the other samples discussed in the remainder of this paper. Figure 4d shows the local histogram of σ_*ii*_ magnitude, which is another way to represent the same data shown in the stress map (c). Abnormal grain growth and surface roughness can be immediately observed in the electron micrograph in (a). On the other hand, the shallow CL probe vividly captures the stoichiometric and micromechanical features of the biomaterial surface, as they arise from both grain-boundary structure and surface polishing. Only a moderate amount of oxygen vacancies is found on the surface, although accompanied by hot spots of residual stress with high magnitude. Average stress histograms collected on larger (*i.e*., statistically meaningful) surface areas of biomedical Al_2_O_3_ materials are shown in Figure 5. The tested materials belonged to two different generations of Al_2_O_3_ bioceramics for hip joints. These histograms show how critical could be even small variations in grain size on the statistical distribution of surface residual stresses. It should be noted that the most detrimental part of the shown stress histograms is the extreme wing on the tensile side. Although it just relates to a quite small fraction of abnormally grown grains in the microstructural network, its impact on wear resistance could be quite pronounced with triggering grain detachment and third-body wear. A successful manufacturing process for an Al_2_O_3_ ceramic hip component should thus be capable to eliminate abnormally grown grains and, thus, to produce a polycrystal with sharp histograms of grain size centered at low-magnitude (surface) residual stresses. Reductions in the average particle size, a tighter control of its distribution, and the improved purity characteristics have been greatly beneficial in obtaining finer microstructures in the latest generation of biomedical Al_2_O_3_. Moreover, with allowing full densification while minimizing sintering temperature (and thus grain growth), the hot-isostatic pressing process introduced in manufacturing the latest generation of biomedical alumina has played a crucially positive role on structural behavior [91]. Provided that cleaning of the bearing surfaces could be conducted with using substances that do not alter surface stoichiometry (e.g., distilled water rather than acetone or ethanol) as is the case here, the CL technique can also be used to visualize the status of worn Al_2_O_3_ joint surfaces after exposure to *in vivo* environment. Our previous studies of retrieved alumina heads from alumina-on-alumina hip joints manufactured by different makers showed that the macroscopic geometry of zones worn to different grades was very different not only among different makers, but also among individual retrievals [14,92].

**Figure 6:**
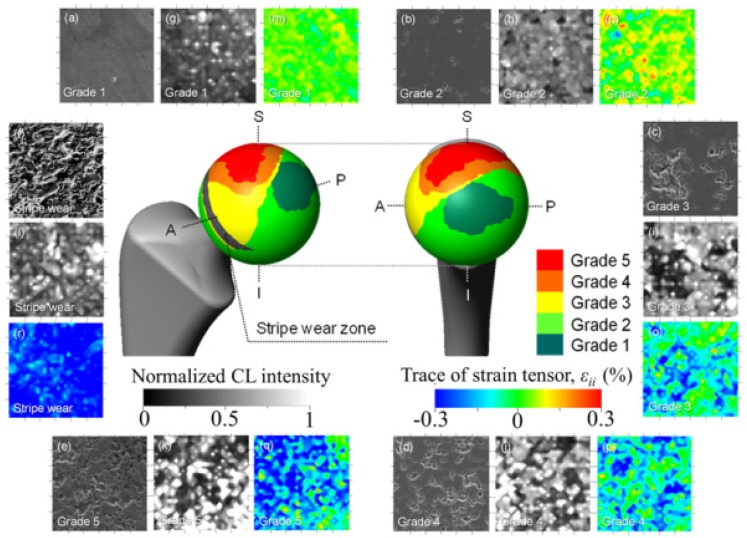
A macroscopic map of surface wear levels on a retrieved monolithic alumina-on-alumina femoral head retrieved after an *in vivo* period of 10.1 yr. Images in inset represent (**a**–**f**) topological; (**g**–**l**) stoichiometric; and (**m**–**r**) micromechanical features for *in vivo* worn areas with different grades of wear severity.

It could generally be stated that the long axis of the ellipsoidal wear shape reflects the direction of primary sliding motion (*i.e*., gait motion) generated at the contact surface of artificial hip joints during *in vivo* loading. However, implant design, liner angular positioning during surgery, and patient range of motion greatly affect local loading characteristics on the bearing surface, which makes predictions of worn surface morphology almost erratic. Figure 6 shows a macroscopic map of surface wear levels on a retrieved monolithic alumina femoral head operating in a hard-on-hard implant (BIOCERAM, distributed by Kyocera Co., Kyoto, Japan). This femoral head was 28 mm in diameter, and belonged to a left (alumina-on-alumina) hip joint, an implant retrieved for aseptic loosening after an *in vivo* period of 10.1 yr. Unused femoral heads of the same type have an average grain size of 1.3 μm and total impurity content <0.2%. The levels of impurity were (SiO_2_+CaO+Na_2_O) < 0.03 wt%, with a content of MgO < 0.01 wt%. Wear severity on the retrieved femoral head was classified into 1~5 Grades of increasing surface damage, defined according to the general statements given by Shishido *et al*. [93]. Surface analyses were performed with great care and high spatial resolution in the scanning electron microscope. However, the Shishido criteria of surface analysis might leave some issues open on the possibility of bias/subjectivity in wear severity assessments.

**Figure 7:**
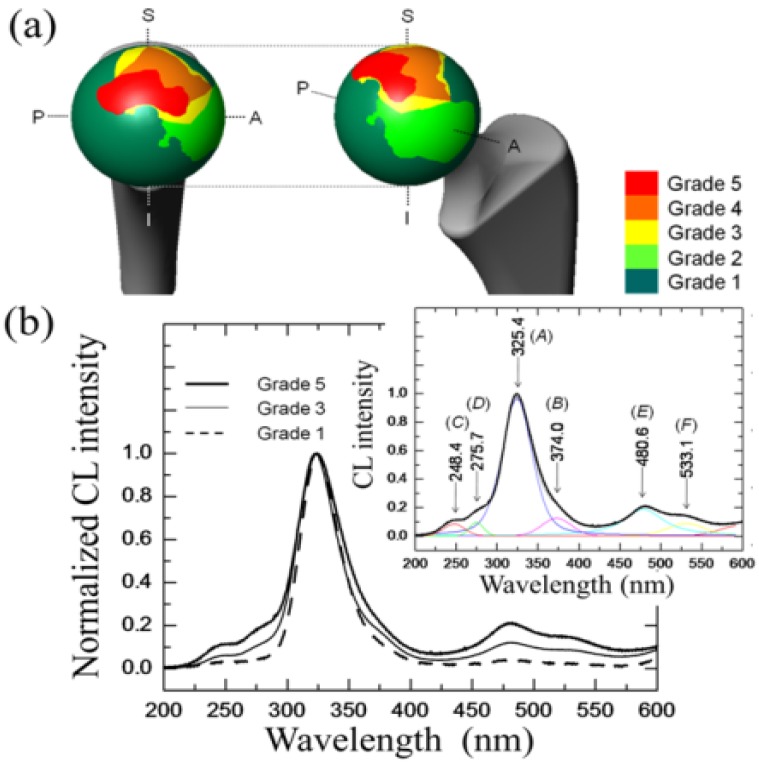
(**a**) Wear severity map for a retrieved monolithic alumina-on-alumina Al_2_O_3_ bioceramic femoral head exposed *in vivo* for 7.7 yr; In (**b**), the increasing presence of aliovalent cations in the Al_2_O_3_ lattice is revealed by means of the CL bands labeled (*C*) and (*D*), which were not found in as-received femoral heads of the same type. Emissions from F^+^ and F centers are labeled (*A*).

In the wear-zone map, a peripheral stripe-wear zone could clearly be found. The wear-zone map revealed that Grades 3 and 4 spread peripherally from Grade 5, while a long and narrow zone of stripe wear, namely the most disruptive damage observed among the articulating regions of the bearing surface, could also be found. This latter region was characterized by a total loss of alumina grains from the original polishing surface, which led to an increase of surface roughness. Images in inset to Figure 6 reveal microscopic aspects of topological, stoichiometric, and micromechanical nature for *in vivo* worn areas with different grades of wear severity. Typical electron micrographs are shown together with maps of concentration of oxygen vacancies (*i.e*., the intensity of the CL band centered at 325 nm and corresponding to F^+^-centers) and of residual (elastic) lattice strain, ε_*ii*_ (*i.e*., as obtained from the σ_*ii*_ magnitudes retrieved from CL spectral-shift analysis of the F^+^ band). The ε_*ii*_ values represent the trace (or hydrostatic part) of the overall strain tensor. Starting from stoichiometric considerations, the topological concentration of oxygen vacancies on the alumina joint surface systematically increased with increasing wear severity, namely with successively progressing from Grade 1 toward Grade 5. The stoichiometric trend found for Grade 0 (*i.e*., an unused femoral head of the same type) was almost indistinguishable from Grade 1 in Figure 6. Even at low wear-severity grades, locations of high concentration of F^+^ centers could be found, not only at grain boundaries, but also in the bulk of the Al_2_O_3_ grains. This finding confirms the important role of surface hydroxylation on vacancy formation in the lattice of biomedical Al_2_O_3_ grades. Areas from the pristine surface increasingly incorporated vacancy sites, but nearly stoichiometric zones (*i.e*., dark areas) were developed at newly exposed locations where grain detachment progressively occurred. However, there is an exception to the observed trend of concurrent increases of wear severity and oxygen-vacancy concentration. The exception resides in the topological frequency of vacancy sites found in the stripe-wear zone. Such frequency was clearly lower than that of Grade 5 and comparable to Grade 2 zones. Note that the statistical presence of oxygen deficient sites, less frequent in the stripe-wear zone than in Grade 5 worn zones, confirms the already established notion that grain removal from the areas of stripe wear is mechanically rather than chemically driven. Another main feature found in comparing the microscopic strain maps in Figure 6 is an increasing shift toward residual compression strains (blue color) in the Al_2_O_3_ microstructure with increasing wear severity (*i.e*., from Grades 3 to 5). Interestingly, however, the elastic lattice strain detected in surfaces classified Grade 1 and 2 was increasingly tensile (red color), and turned toward the compressive side only starting from Grade 3 damage; namely, in correspondence of the beginning of grain detachment from the worn surface. Tensile strain represents what we anticipated as being an exacerbation of pristine tensile stresses at grain boundaries (cf. Figure 4c). The stress data shown in Figure 5 in the histogram for average grain size of 1.3 μm represent the trend found for Grade 0 (*i.e*., an unused femoral head). Such exacerbation is a consequence of *in vivo* environmental effects of chemical nature. A tendency to lattice contraction, associated with the formation of oxygen vacancies, is counterbalanced by the constraint operated by the stoichiometrically unaltered sub-surface. As a result, the surface keeps its original dimensions but undergoes a tensile stress state of chemical nature [94]. Grain detachment is then a consequence of local grain-boundary microfracture, which takes place at *in vivo* regimes of mild wear. At such stage, grain boundaries are weakened and damaged by the tribochemical attack, through processes that in turn lead to exacerbation of pristine tensile strain fields at grain boundaries (*i.e*., those strain fields stemming since material manufacturing). The maximum compressive strain (*i.e*., ε_*ii*_ ≅ 0.3%) was found on the surface of the stripe-worn zone. In a previous paper [95], we have discussed the morphology of the residual stress distribution along the subsurface of a worn hip-joint surface made of Al_2_O_3_. Static equilibrium requires a steep stress gradient to be developed along an axis perpendicular to the joint surface. Stresses along the subsurface thus become of an opposite sign as compared to those found at the surface [95]. This steep stress gradient, actually a measure of the micromechanical instability of the bearing surface, is the main responsible for grain detachment and severe wear damage. As far as F^+^-defect centers and related strain assessments on the microscopic scale are concerned, very similar trends were found for another leading Al_2_O_3_ bioceramic, according to a similar classification of wear severity. In Figure 7a, the wear severity map is shown for a retrieved femoral head belonging to an alumina-on-alumina joint manufactured by a different maker (BIOLOX^®^*forte*, manufactured by CeramTec GmbH, Plochingen, Germany and distributed by Cremascoli Co., Milan, Italy). This BIOLOX^®^*forte* femoral head belonged to a left hip joint and was exposed *in vivo* for 7.7 yr. It was retrieved due to dislocation and not as a consequence of material failure. The impurity contents found in unused BIOLOX^®^*forte* (control) femoral heads were: (SiO_2_+CaO+Na_2_O) < 0.05 wt% and MgO < 0.25 wt%. A comparative wear resistance study of both BIOLOX^®^*forte* and BIOCERAM ceramic-on-ceramic implants, conducted in hip simulator, showed similar results in terms of wear rate and weight loss [96]. As can be seen, the macroscopic geometry of zones worn to different grades was very different from that shown in Figure 6. As compared to Figure 6, Grade 5 zone was located closer to the polar region, Grade 1 zone was quite extensive, and no stripe wear appeared. However, judging about macroscopic wear topology is not the focus of this paper. Moreover, we do not have enough data to comprehensively discuss the suitability of different designs, although a few more topological analyses for similar hip implants have been shown elsewhere and gave consistent results [8,92]. In focus here is the physical chemistry of the *in vivo* exposed alumina surfaces and, in this specific context, a wealth of information could be obtained. A striking feature in investigating the topology of lattice defects on worn Al_2_O_3_ surfaces was that bands related to divalent impurity cations, labeled (*C*) and (*D*) in the CL spectrum of Figure 7b, could be found. While distinctly located with respect to the oxygen-vacancy F^+^/F doublet, these additional bands were systematically stronger at grain boundaries as compared to bulk grains. Cation-related bands were conspicuously absent in virgin samples, and their relative intensity with respect to the band from F^+^ center gradually increased with increasing wear severity after exposure *in vivo*. An increasing presence of divalent cations in the bulk alumina lattice of mildly worn surfaces can thus be interpreted here as a proof for tribochemical interactions between alumina lattice and body environment. Relevant to the present study are divalent cations (e.g., Ca^2+^ and Mg^2+^) that exist as impurities in the raw powders and are also abundantly present in the body environment. As discussed in the previous section, such cations might substitute for Al^3+^ forming a complex F_c_-center, namely an F^+^-center adjacent to a divalent cation site. F_c_-centers of divalent cations emit individual CL bands located in a relatively narrow UV wavelength interval toward 300 nm [73]. Figure 7b shows the increasing presence of such aliovalent cations in the Al_2_O_3_ lattice, whose concentration is directly represented by the relative intensity of the CL bands labeled (*C*) and (*D*) with respect to F^+^ and F centers (bands labeled (*A*) and (*B*), respectively, in the deconvoluted spectra of Figure 7b. Moreover, the doublet emission in the wavelength interval between 450 and 600 nm, which was observed on the worn BIOLOX^®^*forte* surface (cf. bands labeled as (*E*) and (*F*) in Figure 7b), represents the formation of an MgAl_2_O_4_ spinel phase with higher ductility than Al_2_O_3_ [76]. Interestingly, a comparison between this work and Ref. [76] proves a striking similarity in the tribological behavior between Al_2_O_3_ hard-on-hard hip bearings and Al_2_O_3_ cutting tool surfaces after machining steel.

The controversial role of plastic deformation in the wear behavior of Al_2_O_3_ ceramics, as a consequence the adsorption of water at its surface, is an issue already amply discussed in literature [97,98]. Kalin *et al.* [99] have shown that, in extreme pH environments, the main effect controlling wear rate is the dissolution of alumina lattice. This effect leads to wear rates enhanced by one order of magnitude. Moreover, Castaing *et al.* [100] showed that flow stresses in sapphire reduced by a factor of two, due to the presence of water in the lattice, as a result of enhanced dislocation mobility. Concurrently, also grain boundaries in polycrystalline Al_2_O_3_ were found significantly weakened by water. Our CL studies showed major changes in lattice stoichiometry (*i.e*., increase in oxygen vacancies and incorporation of sub-valent cations in the faulted lattice), which could be equally important in enhancing surface degradation under regimes of mild wear. CL spectra from worn Al_2_O_3_ surfaces also suggested the formation of a different phase (MgAl_2_O_4_ spinel) upon tribochemical loading. In other words, our spectroscopic findings emphasize the chemical aspects of surface degradation. The overall evidence converges into a view of an alumina lattice intrinsically limited in its tribochemical resistance in the human body, and thus prone to microscopic degradative wear mechanisms. The degradation of Al_2_O_3_ bearing surfaces *in vivo* is thus basically tribochemical in nature. Accordingly, the hope for an elongated lifetime of monolithic alumina hard-on-hard bearings, as compared to the corresponding hard-on-soft ones, appears to have limited physical foundations. On the other hand, our spectroscopic evidence suggests the possibility of a procedure based on CL spectroscopy for the systematic search of suitable aliovalent dopants enhancing the wear performance of the alumina lattice (a topic discussed in the next section). However, it is also understood that the drawbacks associated with a basic tendency to hydroxylation and to the marked thermal expansion anisotropy of the corundum structure will be hard to overcome.

#### 3.1.2. 3Y-TZP Bioceramics

In order to overcome the poor mechanical behavior of Al_2_O_3_ ceramics, researchers started looking for alternative biomaterial bearings. Pioneering attempts at developing 3Y-TZP as a structural biomaterial were actually based on considering it as a better alternative to Al_2_O_3_ ceramics. This concept was already presented in early papers by Rieth *et al.* [101] and Christel et.al. [102]. A massive industrial development followed, since the middle of the 80s, in the attempt of manufacturing femoral heads capable of overcoming the intrinsically poor mechanical properties of alumina ceramics. Explicit evidence of this vast industrial development in Japan remains attested in a paper by Tateishi and Yunoki from the early 90s [103]. Outside Japan, earliest developments focused on magnesia-partially stabilized zirconia materials, but most of the successive developments were focused on 3Y-TZP, the ceramic alloy nowadays considered as the golden standard in zirconia microstructures. 3Y-TZP consists of a homogeneous network of equiaxed submicron-sized grains that can partly be stabilized in their tetragonal polymorph with grain sizes easily confined to <0.4 μm. Since the 90s, 3Y-TZP has been considered as the standard material for clinical applications [104]. Note that using 3Y-TZP indeed relieves several shortcomings related to the use of alumina bioceramics. For example, a higher crystallographic symmetry is found in its tetragonal structure as compared to the asymmetric hexagonal (corundum) structure of Al_2_O_3_. This property improves the statistical degree of crystallographic coherency at grain boundaries and also minimizes the grain-boundary residual stresses arising from thermal expansion anisotropy. Both these circumstances are beneficial to the macroscopic material strength. Moreover, fully dense 3Y-TZP ceramics can be obtained at sintering temperatures few hundreds of degrees centigrade lower than alumina. Through introducing a post-sintering (low-temperature) hot-isostatic-pressing cycle, dense, fine and homogeneous 3Y-TZP microstructures can indeed be promptly obtained with an average grain size in the order of few hundredths of nanometers and a quite sharp grain-diameter histogram. Note that such microstructural characteristics have definitely proved unfeasible for sintered (monolithic) alumina. However, for the apparently “perfect” family of 3Y-TZP biomaterials, a severe shortcoming has later appeared, which deals with its environmental stability. The fraction of partially stabilized tetragonal phase in the 3Y-TZP sintered body, to be retained at room temperature after cooling from manufacturing temperature, strongly depends on grain size and on the uniformity in concentration of the yttria stabilizing oxide throughout the microstructural network. These factors are also crucial for the response in term of polymorphic stability of the 3Y-TZP biomaterial when embedded in biological environment. In other words, the *in vivo* behavior and the reliability of 3Y-TZP ceramics are strongly affected by manufacturing accuracy and rely on a quite delicate equilibrium among several microstructural parameters (e.g., dopant dispersion, off-stoichiometry state of the starting powder, sintering temperature, *etc.*). The effect of even slight fluctuations in such parameters can inflict a decisive blow to the performance of zirconia as a hip-joint component. In our view, the most decisive parameter in manufacturing reliable 3Y-TZP biomedical components is the control of material stoichiometry from the raw powder to the final sintered body. Therefore, one should hardly trust and could not expect any success from empirical manufacturing procedures unable to strictly assess and to control such parameter in a quantitative way. Note also that the level of stoichiometry fluctuations and the involved microstructural changes, which are critical to the stability performance of 3Y-TZP, are usually considered within standard levels and thus acceptable for alumina ceramics. This is the main reason why, historically, the biomedical market has been keener to develop monolithic Al_2_O_3_ rather than monolithic 3Y-TZP for hip-joint components. Even leaving aside historically tragic examples of failures for 3Y-TZP ceramic implants [105], *in vivo* phase transformation involves detrimental effects on the wear behavior of the polyethylene sliding-counterpart. Serious concerns arose in the past on the effect of sterilization of 3Y-TZP femoral heads in water vapor. This process was found to have an impact on surface roughness due to the tetragonal-to-monoclinic phase transition. In classifying the causes of a number of hip revision surgeries due to osteolysis, surface degradation of retrieved zirconia femoral heads was found to coincide with high wear of ultra-high molecular weight polyethylene sockets [106]. Remarkably, it was found that the femoral heads showing the strongest surface degradation were those very same heads that, although supplied in their sterile status to the hospitals, were re-sterilized in steam (*i.e*., the same autoclave cycle that we use nowadays to test environmental resistance) before implantation. Kawanabe *et al.* [107] measured polyethylene wear radiologically in 46 hips with alumina heads and 58 hips with zirconia heads. In all cases, the preoperative diagnosis was osteoarthritis. The mean linear wear rate of polyethylene sockets against zirconia heads was 0.133 mm/yr, significantly greater than the wear rate of 0.078 mm/yr measured for polyethylene against alumina heads. Wear rates were independent of age at operation, patient body weight as well as height, thickness of polyethylene, and socket abduction angle. The excessive polyethylene wear was then attributed to phase transformation of zirconia, leading to an increase in surface roughness of the femoral head.

A major discovery in biomaterial development has been the beneficial effect of a small addition of Al_2_O_3_ in sintering 3Y-TZP. The physicochemical origin of this effect has comprehensively been summarized in recent papers by Guo [108,109], and it is also of stoichiometry nature. The benefit in adding a small amount (typically a fraction of 0.25 wt%) of Al_2_O_3_ to 3Y-TZP is twofold: (i) an easier densification (*i.e*., meaning a lower sintering temperature and, thus, a finer microstructure) [110,111]; and, (ii) a better environmental resistance in water-vapor environment [112]. However, it might prove difficult to solve problems related to the heterogeneity of such small alumina quantities when mixing with the 3Y-TZP raw powder, due to a generally strong tendency to agglomeration of raw nanometer-sized powders [113]. Again, manufacturing complexity and related increases in costs became critical as compared to the more, stoichiometrically speaking, “unpretentious” alumina.

**Figure 8:**
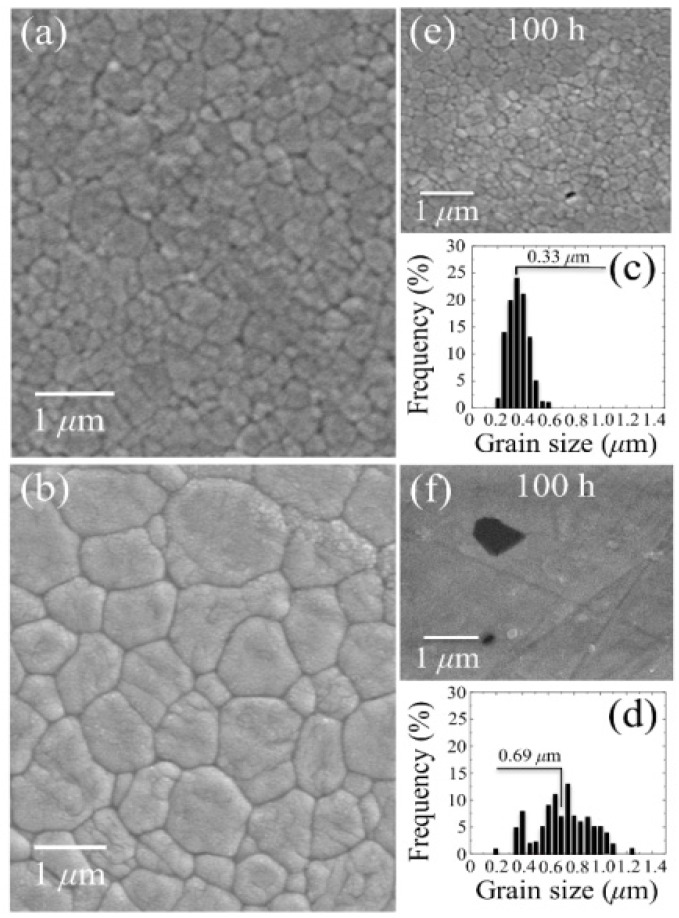
Scanning electron micrographs are shown in (**a**) and (**b**) of the as-received (thermally etched) microstructures of 3Y-TZP Samples A and B, respectively; The respective histograms of grain size are shown in (**c**) and (**d**) (average grain-size values given in inset); In (**e**) and (**f**), surfaces (without thermal etching) after 100 h exposure to hydrothermal environment are given for Samples A and B, respectively.

**Figure 9:**
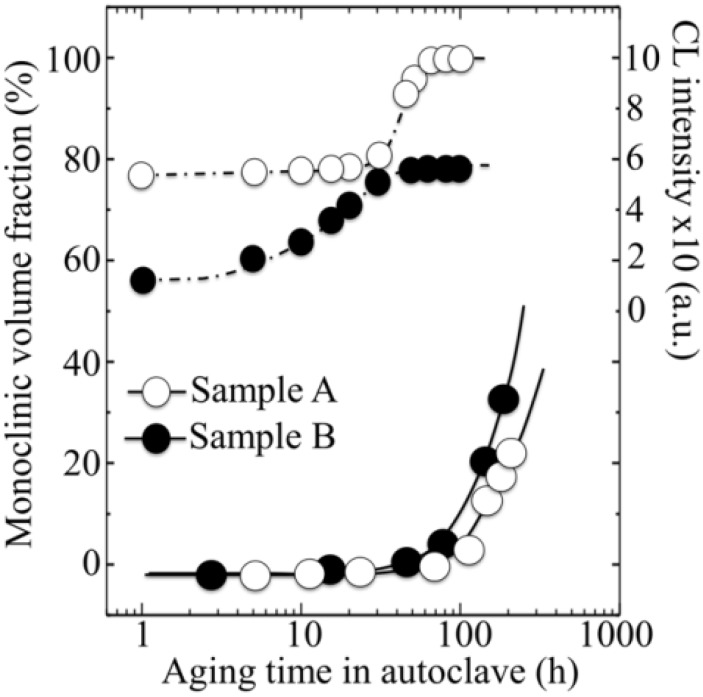
Semi-logarithmic-scale plots of monoclinic phase fractions and amount of annihilated oxygen vacancies as a function of exposure time in hydrothermal environment. The size of the used symbols corresponds to the standard deviation of the measured parameters.

So, if the weak point of 3Y-TZP actually resides in the difficulty of operating a strict control of the off-stoichiometric state of its surface, one should be able to monitor through CL analyses the actual level of accuracy achieved in manufacturing monolithic 3Y-TZP components. Four unused femoral heads for each of two types of commercially available (and most advanced) monolithic 3Y-TZP artificial hip implants were examined with respect to their hydrothermal stability and surface stoichiometry characteristics. The 3Y-TZP samples were 26-mm-sized femoral heads manufactured by Japan Medical Materials (JMM) in 2007 (simply referred to as samples A, henceforth) and 22-mm-sized femoral heads manufactured by NGK in 2007 (henceforth referred to as samples B). Despite nominally having the same composition (*i.e*., both including a fraction of 0.25 wt% of Al_2_O_3_ dopant), the two investigated 3Y-TZP samples distinctly differed in their average grain size (~330 nm and ~690 nm for samples A and B, respectively); a characteristic that is probably due to the different schedules adopted in sintering the two biomaterials. Samples were cut into several pieces and each piece separately subjected to hydrothermal cycles of different duration and temperature in the same autoclave chamber. Hydrothermal acceleration tests were conducted at a temperature of 121 °C under 2 bar water-vapor pressure for periods of time ranging between 0 and 500 h. The hydrothermal tests simulated *in vitro* the effect of environmental aging in the human body. Figure 8a,b shows scanning electron micrographs of the as-received (thermally etched) microstructures of Samples A and B, respectively. The respective histograms of grain size are also shown in Figure 8c,d, together with the average grain-size values given in inset. Figure 8e,f show the surfaces (without thermal etching treatment) of Samples A and B after 100 h exposure to hydrothermal environment under conditions as above. Besides the lower average value of grain size in Sample A and its narrower grain-size distribution, another important morphological feature could be revealed in comparing the microstructures of the two zirconia materials. This additional feature deals with the distribution of the Al_2_O_3_ dopant within respective the microstructures. In the case of Sample B, Al_2_O_3_ grains could be distinctly observed as a dispersion of isolated grains (e.g., the dark grain visible in Figure 8f). On the other hand, no presence of Al_2_O_3_ could obviously be resolved in Sample A (cf. Figure 8e). Grain boundaries in the autoclaved Sample A could easily be visualized because partly “etched out” by the environmental exposure, unlike the smooth surface preserved in Sample B after autoclaving (cf. Figure 8f). In Figure 9, semi-logarithmic-scale plots of both fractions of monoclinic phase and amount of annihilated oxygen vacancies (*i.e*., from average CL intensity trends recorded on the surfaces) are shown, which were detected as a function of exposure time in hydrothermal environment. The plots are similar and comparatively show that the vacancy-annihilation process is precursor to polymorphic transformation. The threshold for steep rising of phase transformation corresponds in both cases to the saturation of the CL plot, which means that oxygen vacancies are fully annihilated at the surface when polymorphic transformation starts to take place. For both Samples A and B, the time necessary for nucleation of the monoclinic phase on the surface of the femoral head is thus preponderant as compared to the time needed for monoclinic nuclei to growth. However, Sample B experienced a nuclei formation rate faster than Sample A at 121 °C. Note that the sigmoidal morphology of the semi-logarithmic-scale plots, which represents the amount of vacancy annihilation with autoclaving time, is clearly different between Samples A and B. As mentioned above, both trends of vacancy annihilation actually saturate before the beginning of exponential rising in the plots of monoclinic contents.

However, comparing the evolutional trends for the respective amounts of oxygen vacancy annihilated, it can also be noted that, for Sample A, a longer nucleation time for the formation of monoclinic nuclei on its surface then corresponds to a faster nuclei growth. Accordingly, the curves giving the transformed fractions as a function of autoclaving time look similar. But, one now needs to explain why Sample A starts picking oxygen from the environment later than Sample B.

**Figure 10:**
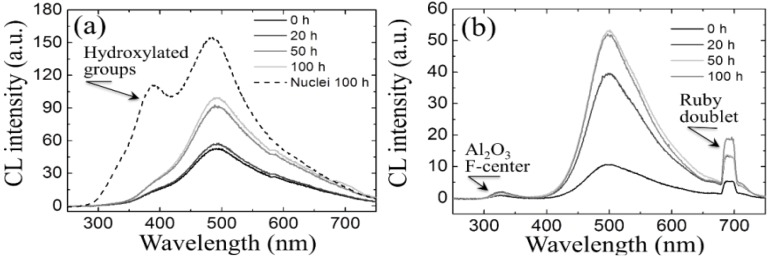
In (**a**) and (**b**), average CL spectra for 3Y-TZP Samples A and B, respectively, which were collected during increasingly long exposures in autoclave up to 100 h.

**Figure 11:**
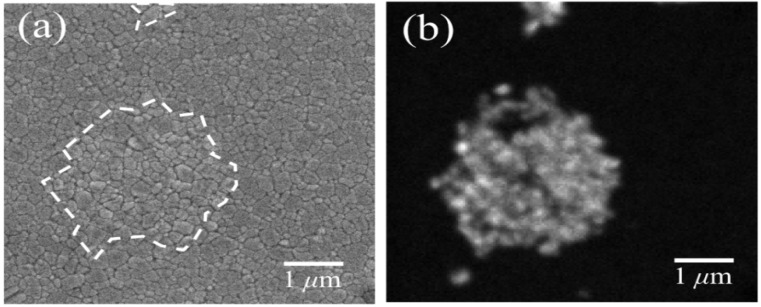
In (**a**) and (**b**), a scanning electron micrograph and a CL intensity maps at 390 nm, respectively, as recorded at the same site on the surface of 3Y-TZP Sample A.

The details of the CL spectra could give us further insight to locate some inherent difference between femoral heads of type A and B in terms of the ionic disorder of their surfaces. Figure 10a,b show average CL spectra (for Samples A and B, respectively), which were collected before and after exposures in autoclave for increasing times up to 100 h. Several striking differences could be found between the spectra of the two samples, as follows: (i) the almost complete absence of the chromophoric CL bands (at around 690 nm) belonging to Al_2_O_3_ in Sample A, which is instead clearly visible in Sample B. The chromophoric emission (or ruby doublet) displays at around 692 nm and 694 nm and stems from native Cr^3+^ impurities in the crystalline alumina lattice; (ii) Sample B clearly showed CL emission from oxygen-vacancy sites in the Al_2_O_3_ dispersoids (*i.e*., the F^+^-center of Al_2_O_3_ emitting at around 330 nm). Note that a failure in detecting CL bands from Al_2_O_3_ in Sample A is not a consequence of a lack of resolution of the CL probe. Rather, it is a proof that the originally added Al_2_O_3_ is in a different physicochemical state as compared to crystalline Al_2_O_3_ in the final (sintered) femoral head component. The lack of dark Al_2_O_3_ grains and, accordingly, of the CL bands characteristic of the Al_2_O_3_ phase in the CL spectra of Sample A, suggests that this sample contains Al_2_O_3_ as a finely dispersed phase in the form of such small grain-boundary precipitates that they might conceivably stem in a molecular-sized (semi-amorphous) state. This is the reason why they emit quite low or no CL signal. This interpretation is also consistent with the fact that grain boundaries in Sample A seem to “etch out” in hydrothermal environment (cf. Figure 8e), while they remain fairly intact upon autoclave exposure in Sample B; (iii) as far as the CL emission from the ZrO_2_ lattice is concerned, the spectra recorded in the two types of femoral head show a common feature in a main broad band, which represents the sum of three distinct emissions from oxygen-related defects with different structures. However, only peculiar to polymorphically transformed zones in Sample A is the appearance of a pronounced shoulder located at around 390 nm (3.18 eV). This shoulder-band was particularly strong in highly transformed monoclinic nuclei on the surface of the femoral head A, as shown in Figure 11a,b which display a scanning electron micrograph and a CL intensity maps at 390 nm, respectively, as recorded at the same site on the surface of Sample A. Whereas scanning electron micrographs conspicuously fail in clearly locating transformation sites, CL emission at 390 nm provides a clear picture of early-stage transformation nuclei on the sample surface. The origin of the local CL emission at 390 nm might also provide a hint about the elementary mechanism behind phase transformation. A plausible interpretation of the 390 nm band could be given according to a study by Nasu *et al.* [114], who attributed this emission to the presence of O-H bonding in an amorphous Al_2_O_3_ structure. Note that an idealized model for fully hydroxylated α-Al_2_O_3_ (0001) foresees the replacement of each surface Al with three H atoms, yielding a coverage >15 OH per square nanometer [38,115]. Hydroxylation of an Al_2_O_3_ structure conspicuously lacking of a crystalline structure should be faster than that of a perfectly crystalline lattice. There should thus be a direct link between the morphological/crystallographic status of the alumina dopant, the degree of ionic disorder in it, and the threshold for destabilization of 3Y-TZP. Hydrothermal dissociation of the Al_2_O_3_ dopant is energetically more favorable, and thus occurs faster than direct vacancy annihilation in metastable 3Y-TZP. This could be the reason why the presence of alumina “protects” the zirconia phase and delays its polymorphic transformation. However, upon saturation of the hydroxylation process of the Al_2_O_3_ phase, its protective function disappears and monoclinic nuclei are formed. Preferentially hydroxylated zones might stem as residual agglomerates since the initial manufacturing step of powder mixing and should be a direct consequence of both powder mixing procedure and the relatively low sintering temperature of Sample A. CL experiments reveal the presence of hydroxylated areas in a straightforward manner, thus indirectly visualizing the peculiar mechanism behind monoclinic nuclei formation in Sample A, an otherwise extremely resistant microstructure to hydrothermal transformation. Summarizing the situation, CL experiments proved that vacancy annihilation on 3Y-TZP surfaces is the main stoichiometric alteration that accompanies phase transformation. However, polymorphic transformation was not simply governed by a monotonic (average) annihilation rate of oxygen vacancies over the entire surface, but stoichiometric alterations were locally driven by the chemical and morphological state of the Al_2_O_3_ stabilizing phase. Increasing the sintering temperature not only promotes ZrO_2_ grain growth, but also makes the alumina dopant precipitating into isolated grains (*i.e*., leaving large areas depleted of Al_2_O_3_ dopant).

This is the case of Sample B, in which a faster polymorphic transformation was detected. Lowering the sintering temperature involves a finer microstructure, but also leaves in the material areas in which the Al_2_O_3_ dopant is easily hydroxylated. Hydroxylated Al_2_O_3_ areas could either form in hydrothermal environment or even survive in the sintered body as agglomerations since mixing of the raw powders. Such areas become potential nuclei from which polymorphic transformation originated and spread on the sample surface. In other words, CL data confirmed how hard could be the task of optimizing sintering temperature in 3Y-TZP: low sintering temperatures keep fine the microstructural array and homogeneous the distribution of stabilizing Al_2_O_3_ phase, but also allow for hydroxylated powder locations to exist as preferential sites for polymorphic transformation. All in all, our data demonstrate how sensitive are biomedical 3Y-TZP commercial grades to all the steps of the manufacturing process, owing to a strong dependence of their environmental resistance on local surface stoichiometry. Mastering how to control at a deterministic level 3Y-TZP stoichiometry issues could have, quite conceivably, led to discover some yet unknown family of zirconia ceramics, which could have shown a superior environmental performance. However, the economic side of the biomaterials business world (and, admittedly, also the level of our scientific understanding) was not ready for such a big research challenge. Consequently, developments abandoned complicated stoichiometry issues and were re-directed toward perhaps the most obvious way to obtain stability and toughness in the same biomaterial: mixing increasingly larger fractions of alumina to zirconia.

### 3.2. Stoichiometry Matters

By applying a spectroscopic approach similar to that followed for monolithic materials, the physicochemical processes governing the *in vitro* surface stability in hydrothermal environment of two leading (commercially available) Al_2_O_3_/ZrO_2_ femoral head components have been investigated at the molecular scale. The two investigated composites were designed according to completely different microstructural concepts. It is shown here how such different design choices have led to completely different responses in terms of oxygen sub-lattice stoichiometry, zirconia phase-stability, and residual stress state on the bearing surfaces, as their stoichiometric characteristics evolved under the effect of hydrothermal environment. Moreover, proof is also given of the impact of such responses on the intrinsic wear resistance of the two composite biomaterials.

Al_2_O_3_/ZrO_2_ composites can be considered to be the latest technological development in bioceramics for arthroplastic applications. From the viewpoint of material design, they represent a microstructural “compromise” that could allow preserving both the biocompatibility and the phase stability of monolithic alumina in the human body and the enhanced mechanical properties of monolithic zirconia, through the exploitation of its peculiar mechanism of transformation toughening [116,117]. This simple material design concept has indeed been shown to work properly and Al_2_O_3_/ZrO_2_ composites have been reported to possess significantly improved mechanical properties, including a higher structural reliability, as compared to biomedical (monolithic) Al_2_O_3_ materials. In one of the two composites investigated here (BIOLOX^®^*delta*, manufactured by CeramTec GmbH, Plochingen, Germany; henceforth referred to as Composite A), the reinforcing phase consisted of 17 vol% fraction of sub-micrometric ZrO_2_ particles, which were partially stabilized with ~0.6 wt% Y_2_O_3_, while also a fraction of 0.3 wt% of Cr_2_O_3_ and a minor fraction of SrO phases were added to the raw powder. After sintering, Y and Cr elements were founds mainly solved within the ZrO_2_ and Al_2_O_3_ lattices, respectively. On the other hand, the Sr element was fully incorporated in an SrAl_12−*x*_Cr*_x_*O_19_ (strontium aluminate) compound, which represented about 3 vol% of the final sintered body. Because of its successful clinical history, Composite A has gained unrivaled popularity worldwide in the bioceramic market, which stands since more than 10 years. Recently (since the year 2009), a new Al_2_O_3_/ZrO_2_ composite (KM AZ209, manufactured by Kyocera Medical Co., Kyoto, Japan; Composite B, henceforth) has been launched in the Japanese market [118,119]. This new material contains a 14 vol% fraction of fully unstabilized sub-micrometer-sized ZrO_2_ particles in addition to traces of other oxide additives for a total fraction of 2 vol% (SrO, TiO_2_, MgO, and SiO_2_ with relative fractions undisclosed by the maker). Unlike other cations, Ti ions were confirmed to stem in a solid solution of the corundum Al_2_O_3_ lattice, as better specified later. The microstructure of Composite B was tailored not only for matching the requirement of improved mechanical properties as compared to monolithic Al_2_O_3_, but also for achieving a complete stabilization of the ZrO_2_ phase in the severe environment of the human body. Note that both Composites A and B can be classified as bioceramic materials possessing extremely refined microstructures as compared to (monolithic) biomedical Al_2_O_3_ grades [120]. The mechanical properties published by the respective makers [118,121] were outstanding for both composites, including strength values well above 1 GPa and toughness values in the order of >4 MPa·m^1/2^; thus, far above those reported for monolithic alumina [122]. However, due to the quite recent release on the market of Composite B, a comparison between these two leading bioceramics in terms of clinical performance is still missing in the published literature.

**Figure 12:**
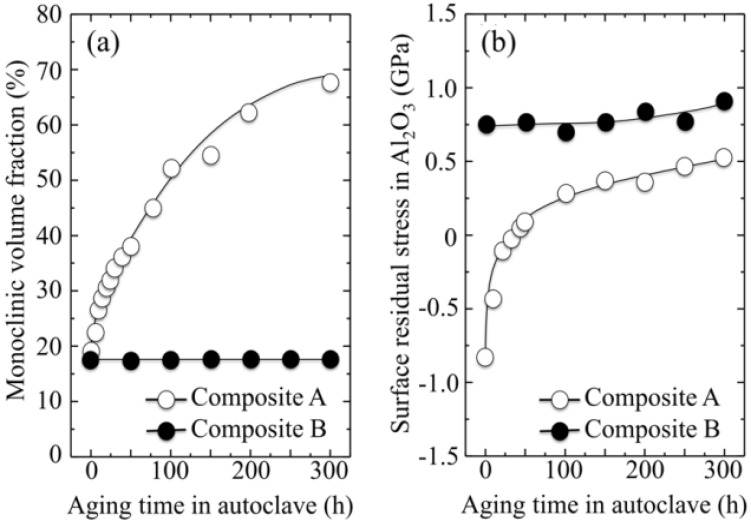
(**a**) Volume fractional evolution of the ZrO_2_ monoclinic polymorph in the microstructure of Composites A and B as a function of aging time in a climate test chamber (the *y*-axis refers to fractions of the overall ZrO_2_ fractions); (**b**) evolution of the residual stress stored onto the surface of the Al_2_O_3_ matrix phase of Composites A and B as a function of aging time in a climate test chamber.

Figure 12a shows the volume fractional evolution of the ZrO_2_ monoclinic polymorph in the microstructure of Composites A and B as a function of aging time in a climate test chamber operating at 121 °C in water vapor. Autoclave exposures were intentionally extended up to very long durations (300 h) in order to scrutiny the ultimate response of the materials to hydrothermal attack. The two studied composites experienced almost the same fractions of monoclinic ZrO_2_ polymorph in their as-received state. In Composite A, the initial monoclinic fraction (~19 vol% of the added ZrO_2_ fraction, corresponding to ~3.2% of the overall material volume) increased up to a saturated value of ~70 vol% of the overall ZrO_2_ fraction (*i.e*., approximately 12% of the overall material volume). On the other hand, exposures in autoclave up to 300 h left the ZrO_2_ monoclinic fraction on the surface of Composite B almost completely unaltered at its initial fraction of ~17.5 vol% (*i.e*., ~2.5% of the overall material volume), as recorded in the as-received femoral head. In other words, the microstructural design of Composite B appears quite successful in fully controlling the environmentally driven development of monoclinic phase on the material surface. Note that, from a purely thermodynamic point of view, such performances *in vitro* indicate a significant progress in the material stability as compared to monolithic 3Y-TZP. If one neglects the effect of lipids, mechanical stresses, and other tribochemical contributions taking place in human joints, such long-term exposures could be estimated to correspond to *in vivo* periods of several human lifetimes when extrapolated to body temperature [123]. However, due to the differences between *in vitro* (no stress, no presence of lipids) and *in vivo* exposures, autoclave experiments are not conservative. Figure 12b shows the evolution of the residual stress stored onto the surface of the Al_2_O_3_ matrix phase of Composites A and B as a function of aging time in a climate test chamber operating as given above. The shown stress magnitudes correspond to the trace of the stress tensor, σ_*ii*_, and represent values averaged over sample depths ≤70 nm. A comparison makes immediately clear how the recorded stress values in the as-sintered status and their successive evolutions are markedly different between the two investigated composites. The Al_2_O_3_ phase on the surface of Composite A was found to lie in a strongly compressive residual stress state in its as-received state, then it experienced a steep increase toward a stress-free condition (*i.e*., up to 50 h exposure in hydrothermal environment), to finally endure tensile stresses up to 500 MPa for any further elongation of autoclave exposure. In Composite B, exposures in autoclave up to 300 h produced only a mild increase on the average magnitude of residual stress stored onto the surface of its Al_2_O_3_-matrix. However, the recorded stress magnitudes, always tensile in nature, were as high as 0.75 GPa since the manufacturing stage. Note that such high magnitude of tensile residual stresses is confined to the very surface of the femoral head. Therefore, such residual stresses do not directly affect the bulk mechanical properties (*i.e*., strength and toughness) of the overall component. Their main effect should be expected on the wear resistance of the material in hard-on-hard couples, as it will explicitly be discussed later in this section. This author believes that systematic measurements of surface residual stresses on the bearing surfaces of ceramic components should mandatorily be performed by medical device industries when designing ceramic microstructures, at least in those components to be employed in hard-on-hard implants. Ignoring the behavior of residual surface stresses and their decisive role in wear resistance might be the results of a long-standing incapacity of quantitatively measuring them. However, CL studies clearly show here the possibility of analyzing surface stresses at the nanometer scale, thus clarifying their interconnection with chemical and environmental parameters.

Now, the challenge shall shift on how to explain such striking differences in surface stability and residual stress behaviors, despite the quite similar compositions of the two investigated composites. It is shown hereafter that the origin of such differences resides in some basic physical chemistry aspects of the composite surfaces. It should also be noted at the outset that the behaviors of the two investigated composites follow a quite counterintuitive trend in the frame of our general understanding of phase stability in ZrO_2_ ceramics. For similar Al_2_O_3_ matrices, unstabilized ZrO_2_ dispersoids (Composite B) seem to be more environmentally stable than Y-stabilized ones (Composite A).

Specifically regarding the residual stress fields, static equilibrium requires that a tensile stress state in the Al_2_O_3_ matrix induce a compressive stress state in the ZrO_2_ grains, and vice versa. Moreover, it is known that compressive stress fields have a stabilizing effect on the tetragonal ZrO_2_ polymorph, while tensile ones destabilize it [124].

The main causes of residual stress in Al_2_O_3_/ZrO_2_ composites might be classified into three categories, as follows: (i) thermal residual stresses, which arise upon cooling from the manufacturing temperature and are caused by a mismatch in thermal expansion between the constituent phases; (ii) phase-transformation-induced residual stresses, which stem from the (constrained) volume expansion that accompanies polymorphic transformation of the ZrO_2_ lattice; and, (iii) compositional (or chemical) residual stresses, which might become significantly large when a strong oxygen off-stoichiometry is accumulated onto the material surface (*i.e*., upon dopant addition and/or through interactions with the environment). Note that thermal stresses (*i.e*., the above type (i)) in the Al_2_O_3_ matrix are expected to be of a compressive nature when the ZrO_2_ phase in the composite is predominantly tetragonal, owing to a lower thermal expansion coefficient of Al_2_O_3_ as compared to tetragonal ZrO_2_ [125]. On the other hand, as far as stresses of type (ii) are concerned, a stress component of tensile nature should pile up in the Al_2_O_3_ phase in order to counterbalance the compressive stress field created in the constrained ZrO_2_ grains upon their transformation into the monoclinic polymorph (*i.e*., accompanied by lattice expansion) [125,126]. Regarding the effect of off-stoichiometry on the stress state of the surface, the rationale has already been given in discussing the trends observed for monolithic Al_2_O_3_. The lattice contraction associated with the formation of oxygen vacancies should potentially lead to surface shrinkage, but the constraint operated by the stoichiometrically unaltered sub-surface keeps the surface at its original dimension. The result is a tensile stress field of chemical origin stored within the faulted Al_2_O_3_ lattice of the bearing surface [94,127]. With these notions in mind, one could deduce that since Composites A and B in their as-received state have similar volume fractions of both tetragonal and monoclinic ZrO_2_ polymorphs, there is no reason why the residual stress components of the above types (i) and (ii) should differ. Moreover, stresses of types (i) and (ii) could by no means experience strongly tensile magnitudes, which were instead detected in Composite B. This is indeed another quite counterintuitive issue, unless one assumes a significant contribution of stresses of chemical origin (*i.e*., the above type (iii)) on the micromechanical state of the composite surface.

**Figure 13:**
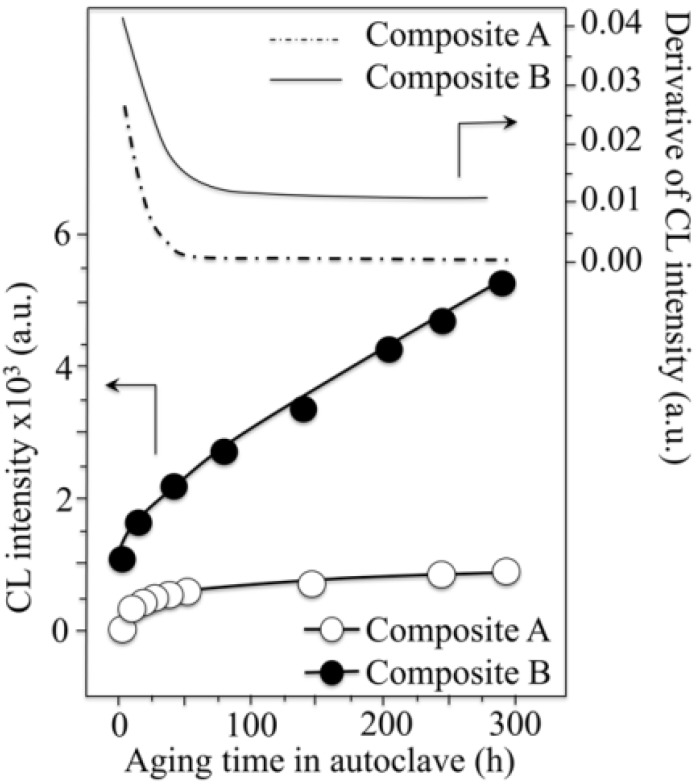
Trends found for CL intensity of bands related to oxygen deficiency in the Al_2_O_3_ matrix as a function of exposure time in autoclave for both Composites A and B. The size of the used symbols corresponds to the standard deviation of the measured CL intensity. The derivatives of the curves are also shown, which represent the vacancy formation rate on the surface.

The trends found for the CL intensity of bands related to Al_2_O_3_ oxygen deficiency are shown as a function of exposure time in autoclave in Figure 13 for both the investigated Al_2_O_3_/ZrO_2_ composites. The first derivatives for the trends are also shown, which give the rate of oxygen-vacancy formation in the Al_2_O_3_ phase. The main off-stoichiometry features related to the effect of environmental exposure can be listed as follows:

(i) The intensity of the F^+^-center emission (at 325 nm) from oxygen vacancies in the Al_2_O_3_ lattice was quite low in the as-sintered Composite A as compared to Composite B, although the intensity of this spectroscopic band consistently increased in both composites with increasing exposure time in autoclave. This means that oxygen vacancies were increasingly (and similarly) created on the surface of both alumina matrices by the effect of hydrothermal treatment. However, such increase in concentration started from quite different initial values (*i.e*., significantly lower in Composite A).
(ii) The derivative plots in Figure 13 show that vacancy formation in the Al_2_O_3_-matrix phase of Composite A tended to saturate (derivative approaching zero), while in Composite B vacancy concentration continuously increased at a constant rate after an initially (mild) exponential rising.
(iii) It was also found that the cumulative intensity of the CL emission from F^+^ and 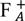 centers in ZrO_2_ (not shown here) strongly increased upon autoclaving Composite A, but remained almost constant (at high intensity) in Composite B up to 300 h exposure. Reminding that the intensity of these CL bands is representative of the concentration of oxygen vacancies annihilated in the ZrO_2_ lattice, one could deduce that the tetragonal ZrO_2_ phase in Composite A was initially rich in vacancies (due to the presence of Y^3+^ in its lattice), but underwent vacancy annihilation upon hydrothermal exposure. On the other hand, the as-received Composite B possessed a lower amount of oxygen vacancies in its ZrO_2_ phase, due to a lack of stabilizing phase. But, such low fraction was stably retained even after quite extended environmental exposures. We know from our studies of monolithic Al_2_O_3_ that the increasing trend for the F^+^ emission from the Al_2_O_3_ lattice is due to surface hydroxylation followed by the formation of oxygen vacancies, in order to maintain electrical equilibrium on the material surface [27]. However, owing to the presence of traces of different dopants in solid solution in the corundum structure, there are fundamental differences between the processes of vacancy formation occurring in the Al_2_O_3_ matrices of the two studied composites. The very low initial amount of oxygen vacancies in the Al_2_O_3_ phase of Composite A (cf. CL intensity at 0 h in Figure 13) can be explained by considering that the Cr^3+^ isovalent cation behaves as a protective element against oxygen defect formation [128]. On the other hand, Ti^3+^ ions do not possess such property, besides their isovalent incorporation into the Al_2_O_3_ lattice should be similarly possible without alteration of the native oxygen sub-lattice [129]. Despite the steep vacancy-formation rate experienced in the first 50 h of autoclave exposure, the intensity of CL emission from the Al_2_O_3_-F^+^ centers in Composite A always remained distinctly below that recorded for Composite B. In other words, there was a systematically higher oxygen vacancy concentration in the latter biomaterial. Being a donor-type dopant, Ti^3+^ sites should tend to annihilate through enhancing the incorporation of oxygen excess in the crystal lattice (*i.e*., with providing the electrons that are required for trapping O^2−^ from the environment). Oxygen vacancies might then gain the outer electron of Ti^3+^ sites creating (Ti^4+^-F^+^) spatially correlated complexes in the Al_2_O_3_ lattice.

In other words, unlike Cr^3+^, when Ti^3+^ is solved in the corundum structure, it tends to change its valence and to favor oxygen vacancy formation more quickly than undoped Al_2_O_3_. High concentration of oxygen vacancies is then the reason for the highly tensile stress magnitudes recorded in the Al_2_O_3_ phase, which thus prove being of a chemical/stoichiometric nature. For obeying static equilibrium, ZrO_2_ dispersoids must then be embedded in a strong compressive stress field, which thus stabilizes them even in the absence of Y^3+^ dopant. It is therefore demonstrated that oxygen stoichiometry in the Al_2_O_3_ matrix is governed by small amount of different dopants (Cr^3+^ *vs.* Ti^3+^) and can lead to different phase-stability behaviors, as found in the studied composites and depicted in Figure 12a.

**Figure 14:**
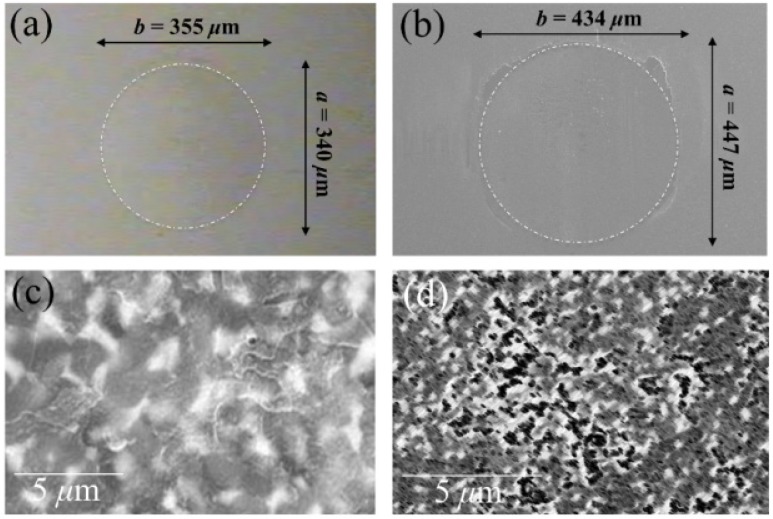
Optical images at the locations of elliptical scars on the pins made of Composites A and B after self-mating tests lasting 2 × 10^3^ m in a pin-on-ball configuration (in (**a**) and (**b**), respectively); In (**c**) and (**d**), high magnification electron micrographs are shown from the damage scar regions (from ball side) in Composites A and B, respectively.

The impact of stoichiometry and residual stress states of the composite surface on the intrinsic wear resistance of self-mating bearings was assessed by means of a specially designed pin-on-ball tribometer, which enabled testing femoral heads as received from the maker. The details of this testing device have been given in previous publications [130,131]. It suffices here to consider that the testing conditions were those of a completely dry sliding and were adjusted for exactly matching the international standards released for the pin-on-disk wear testing configuration (*i.e*., ASTM G99/G95, DIN 50324, and ISO/FDIS 20808:2003(E)). Although dry sliding might be judged as a quite severe condition with respect to standard wear simulations in protein-lubricated environment, the pin-on-ball testing procedure presented here not only allows one to test the ultimate hard-on-hard wear resistance of the material, but could also be a realistic test in simulating a lack of synovial fluid *in vivo* in ceramic-on-ceramic hip joints. The results of a search for elliptical scars after wear tests on the pins made of Composites A and B after testing for 2 × 10^3^ m are shown in Figure 14a,b respectively.

The scar in Composite A was quite shallow as compared to Composite B, and it could not be clearly observed under the optical microscope. The modification of the surface due to wear were resolved by mapping the scar in the SEM, which allowed us to plot the scar diameter as shown in Figure 4a. The diameter of the scar in Composite A was smaller than that of Composite B (cf. scar diameters in Figure 14a,b). Accordingly, the specific wear rate of the former composite couple was about one order of magnitude lower than that of the latter (2.23 × 10^−17^ *vs.* 2.42 × 10^−16^ m^2^/N). The measured friction coefficients were similarly low (~0.38) in both composites as compared to that measured under the same tribological conditions in monolithic Al_2_O_3_ (*i.e*., ~0.52). Testing for comparison monolithic alumina femoral heads gave a specific wear rate of 1.03 × 10^−16^ m^2^/N, thus revealing that the specific wear rate of Composite B in self-mating tests was twice as worse as that of monolithic alumina bearings. Figure 14c,d) show higher magnification electron micrographs taken in damage scar regions (from the ball side) of Composites A and B, respectively. The micrographs reveal a quite high level of surface damage in Composite B, with significant ZrO_2_ grain detachment. On the other hand, the damages on the surface of Composite A were limited to micro-scratches, only quite sporadically accompanied by grain detachments (*i.e*., no grain pullout is indeed observed in the shown micrograph). The behavior of Composite A was quite improved as compared to that of a biomedical grade of monolithic Al_2_O_3_, as shown in details in a previously published report [131]. However, the wear performance of Composite B was even slightly worse than that of monolithic Al_2_O_3_. This suggests that hip joints made of Composite B are not suitable for hard-on-hard bearings or, at least, they do not show any improved wear performance as compared to monolithic Al_2_O_3_. In conclusion, stoichiometry matters in bioceramics. Sophisticated empirical optimizations have led to Composite B, which is probably the most “bioinert” ceramic ever launched in the hip-joint market. However, the wear performance of this new material appears to be disappointing, if the ultimate goal is set to produce hard-on-hard joints lasting as long as a human lifetime. On the positive side, the antithetical effect of Cr^3+^ and Ti^3+^ suggests the possibility of tailoring the surface properties of Al_2_O_3_/ZrO_2_ composites through suitably doping the Al_2_O_3_ matrix phase.

### 3.3. Ionic or Covalent?

Basic notions of chemistry teach us that water can dissolve many ionic compounds because of the polarity of its molecules. The physical chemistry origin of such phenomenon resides in the fact that ionic compounds have a charge and can interact with either side of the water dipole. Such interaction might ultimately lead to breakage, as we have seen, of the ionic molecule by hydroxylation or dissociative chemisorption. On the other hand, (non-polar) covalent compounds cannot form hydrogen bonds and thus are expected to have little or no interactions with water molecules. According to the above reasoning, using covalently bonded ceramics in principle appears to be the most straightforward solution to improve bioinertness in hip-joint bearings. But, are things actually so simple? The need for high toughness and structural reliability would suggest driving our attention on Si_3_N_4_ ceramics as a primary covalent-bonded candidate for arthroplastic joint applications [132,133,134,135]. Toughness of a commercially available Si_3_N_4_ biomaterial (manufactured by Amedica Corporation, Salt Lake City, UT, USA) has been reported as two to three times higher than that of monolithic Al_2_O_3_ and was also appreciably higher than that of the leading Al_2_O_3_-ZrO_2_ composites (Composite A in the previous section), toughened by polymorphic transformation [136]. However, also Si_3_N_4_ bioceramics are polycrystalline in nature and, for manufacturing them, liquid-phase sintering is usually carried out by addition of oxides, including Y_2_O_3_ and Al_2_O_3_ [137,138]. The addition of oxide phases during sintering is a fundamental prerequisite for the achievement of an acicular grain morphology and for the obtainment of intergranular fracture mode, namely the two concurrent requirements needed for achieving high toughness [139,140,141,142,143]. However, the chemical composition of grain boundaries filled with oxide phases might also play a role in long-term bioinertness and contact-damage resistance. Moreover, the bulk lattice, which actually is a Si-Al-O-N structure (with residual Y stemming at grain boundaries) [137,142] contains highly electronegative atoms (*i.e*., nitrogen), which can form H-bonds to the water molecules. Understanding the tribochemical role of such processes represents the key to discuss the suitability of Si_3_N_4_ in hip arthroplasty. The tribological properties of silicon nitride are excellent but, as those of many other ceramics, strongly depend on the specific outputs of tribochemical reactions. Such reactions modify both surface composition and morphology. Fortunately, however, not all the tribochemical reactions have a negative impact on wear. Under humid conditions, hydroxylated silicon oxide is the main product of tribochemical wear in self-mated Si_3_N_4_ bearings, which flattens the surface thus decreasing stresses on asperities and reducing wear rate and friction level. Si_3_N_4_ ceramics can indeed be chemically very stable, although under frictional conditions their surfaces might become quite reactive. Covalent bonds in ceramics are strong due to shared electrons between atoms of similar electronegativity. However, boundary lubrication studies of silicon nitride [144,145] suggested that its reactivity with water was consistent with wear testing results on the effect of humidity on wear rate. The proposed reaction process is based on a regular thermochemical reaction. Taking now into account the present trend toward the incorporation of non-oxide ceramics into joint prosthetic applications, more comprehensive understanding of ceramic thermal chemistry and tribochemistry in biological environment is obviously needed. Available data for wear testing of Si_3_N_4_ in water [146] have shown that after an initial (relatively) high wear rate, water generates a strong lubrication effect with silicon nitride.

To explain this phenomenon, the following chemical reaction had been postulated, which envisage the formation of a “lubricating” layer of silica:

Si_3_N_4_ + 6H_2_O → 3SiO_2_ + 4NH_3_ (5)

**Figure 15:**
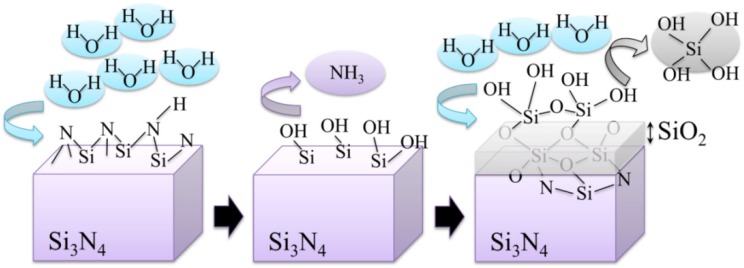
Behavior of a silicon nitride ceramic surface in a tribochemically active environment.

Some paper has described ammonia formation (*i.e*., Equation (5)) from silicon nitride in the presence of water, according to a mechanistic model [147]. The reaction is a mechanically activated hydrolysis by water vapor, *i.e.*, a reaction caused by a direct contact between disturbed Si-N bonds and water, which yields silica and ammonia [148]. Muratov *et al*. [149] introduced the intriguing idea that the cause of the enhancement of the reaction rate (or tribochemical wear rate) in Si_3_N_4_ ceramics might reside in bond stretching that lowers the gap between electronic orbitals, vibrational excitation pulses, and dissipated heat. All these factors are linked to bond stretching and molecular vibrations, which we can measure by spectroscopic tools. It is also emphasized that friction might activate the reaction and this implies also that active reaction sites are produced by frictional work. The mechanism of tribochemical reactions (*i.e*., schematically shown in Figure 15) thus involves subsequent steps of formation of active sites (ammonia, silicic acid, and hydroxylated SiO_2_ condensation products) and adsorption layers, formation of products by tribochemical reaction, and removal of such products (e.g., light molecules, larger molecules, or particles) from the surface. The exact determination of the chemical nature of active sites is indeed a difficult matter since they react during frictional sliding. However, the overall phenomenon, unlike the case of oxide ceramics, does not appear to lead to surface degradation, but rather to a surface self-protective cycle. Note that the tribochemical picture of Si_3_N_4_ fits well into our concept here: deeper knowledge of physical chemistry leads to better bioceramics. Moreover, ammonia is known to annihilate bacteria by raising the local pH to highly alkaline levels where bacteria cannot survive [150]. When dissolved in water, ammonia might capture protons (*i.e*., while becoming charged to NH^4+^), thus reducing proton concentration and locally raising the environmental pH. Therefore, not only Si_3_N_4_ is conspicuously unaffected by the detrimental effects of environmental protons during *in vivo* environmental exposure, but it also possesses the ability to capture protons. This property might represent a therapeutic advantage for osteoarthritic patients. It is known that osteoarthritic inflammation (and subsequent joint damage) is the result of oxidative stress, namely an imbalanced state whereby free radicals might predominate over antioxidants. Oxidative stress is in turn the consequence of multivariate and concurrent factors, including the formation of endotoxins such as those produced from bacteria, dietary factors such as excess sugar and saturated fat, and insulin resistance. Biomechanical stress then enhances structural and biochemical changes within the cartilage, which result in the formation of free radicals and in the occurrence of detrimental chemical reactions. It is suggested that the function of locally neutralizing excess protons, enhancing local pH, and minimizing sources of free radical production could also be one of the advantages of using Si_3_N_4_ ceramics in orthopedic and other biomedical applications.

Similar to the case of ceramics with ionic bonds, also the covalently bonded Si_3_N_4_, in spite of the (wrong) ceramic reputation of chemical inertness, is susceptible to tribochemical reactions, which in turn lead to the formation of surface films and thus modify its frictional behavior. However, as we already stated, not all reactions come to deteriorate the surfaces. As several other non-oxide ceramics, Si_3_N_4_ exposed to air commonly forms an oxide film on the sliding surfaces. The oxidation film can thus react with water, according to the following equation:

SiO_2_ + 2H_2_O → Si(OH)_4_ (6)

The frictional coefficient measured by pin-on-flat standard techniques is strongly affected by such reaction and experiences initial values of ~0.6 (*i.e*., higher than that of monolithic Al_2_O_3_ and Al_2_O_3_/ZrO_2_ composites). However, as the oxidation reaction proceeds, the coefficient of friction of Si_3_N_4_ gradually decreases down to values as low as 0.32 [145,146,147,148,149,151]. In substance, tribochemical reactions in Si_3_N_4_ provide flat surfaces and decreases stresses since insoluble tribo-products might act as lubricants by forming protective films, such as hydrated silicon oxides in water vapor environment. In other words, self-mating Si_3_N_4_ bearings appear to possess a self-lubricating function. Therefore, from the point of view of a bulk Si_3_N_4_ lattice, one could conceivably expect an improved long-term wear behavior in hard-on-hard hip couples. Such improvement could lead to a significantly elongated lifetime as compared to oxide ceramics.

What about the role played by grain boundaries in non-oxide ceramics? We mentioned that sintering of Si_3_N_4_ ceramics up to a fully dense state would necessarily require the addition of a substantial fraction of oxide additives to the starting Si_3_N_4_ powder. Such oxide additives melt at high temperature and form a liquid phase. This phenomenon helps the material to reach a full densification (*i.e*., otherwise hindered by a quite low self-diffusion coefficient), and allows the microstructural development of acicular grains that leads to high toughness and high reliability [139,140,141,142,143]. After sintering, the additive phase-mixture stems at grain boundaries as a continuous film [139,152]. This is the reason why the “wet” Si_3_N_4_ grain boundaries, unlike the “dry” ones of an equally hexagonal Al_2_O_3_ polycrystal, possess a good coherency and reduced residual stresses of thermal origin, even at unfavorable neighboring grain orientations. One challenge, however, might reside in optimizing the role of grain boundary phase with respect to contact damage behavior. This is indeed a crucial issue in hard-on-hard hip bearings, for which sliding is expected to occur under conditions of swing and microseparation. To minimize contact damage, strong grain boundaries might be required, while toughening should instead require substantial interface debonding, and thus weak grain boundaries. This will obviously be a matter of microstructural compromises that have to be searched for in properly setting the material processing conditions. The commercially available Si_3_N_4_ biomaterial manufactured by Amedica Corporation contains a relatively large fraction of Y_2_O_3_ and Al_2_O_3_ phases (6 wt% and 4 wt%, respectively) as sintering aids. This biomaterial is successfully used in spine fusion devices, but is also a primary candidate for hip-joint bearing couples. Preliminary data on Amedica Si_3_N_4_ show quite promising outputs not only in terms of wear resistance and tribochemical bioinertness, but also in terms of new functionalities related to anti-infective and osteointegration properties [153,154]. In other words, the future might be bright for Si_3_N_4_ bioceramics components of hip-joint implants, as they are now clearing up the pre-clinical conditions for becoming the first non-oxide ceramic employed in hip arthroplasty.

## 4. Conclusions

With this paper, we aimed at expressing and documenting the need for entering upon an updated chapter of hip-joint tribology including physical chemistry concepts in the definition of bioceramic inertness and studies of *in vivo* tribochemical phenomena. Clear evidence has been given for tribochemical interactions occurring between body environment and the alumina lattice, new aspects unveiled for zirconia instability and for the role of small amounts of additives in Al_2_O_3_-ZrO_2_ composites. From this body of new physical evidence, it vividly appears how surface off-stoichiometry and micromechanics are tightly related to each other and both governed by the physicochemical behavior of the ceramic surfaces in biological environment. All these phenomena have so far been almost completely neglected, probably simply because there was no an efficient analytical tool or methodology to clearly visualize them. CL spectroscopy proved thus quite innovative in this context, allowing one to locate the concentration of point defects, to map them with high spatial resolution, and to use their stress sensitivity for characterizing so far unknown magnitudes of surface stresses stored onto the ceramic bearing surfaces. It should now be established that the common sense of oxide bioceramics being fully bioinert in the human body is only based on an approximate view of these materials. Moreover, this concept is intrinsically untrue at the molecular scale. The misleading assertion of oxide-ceramic bioinertness has long spread throughout information networks and might have induced even the expert audience to retain inexact understanding. Physical chemistry arguments instead suggest that it could be the time to move forward from oxide ceramics, whose lifetime will necessarily be limited owing to their intrinsic oxygen activity in hydrothermal environment. We need now to abandon obsolete stereotypical views, finding the strength and the determination to explore new alternatives for hip-joint bearing materials. Quite positive news will likely come from the class of non-oxide ceramics.
